# Porous Transducers and Hybrid Recognition for Bulky Nitrosamine Impurities: Toward 3D–MXene–Aptamer Detection of Tricyclic NDSRIs

**DOI:** 10.3390/bios16100535

**Published:** 2026-09-24

**Authors:** Tirath Shah, Ankur Patel, Jignesh Shah, Darshil Shah

**Affiliations:** 1 Department of Quality, Aspire Pharmaceuticals, Somerset, NJ 08873, USA; tirath.shah@aspire-pharma.com; 2 Veeda Clinical Research Ltd., Ahmedabad 380015, Gujarat, India; 3 L J Institute of Pharmacy, L J University, Ahmedabad 382210, Gujarat, India; jignesh.shah_ljip@ljinstitutes.edu.in (J.S.); darshil.shah@ljinstitutes.edu.in (D.S.)

**Keywords:** NDSRIs, N-nitroso clozapine, 3D-printed electrodes, Ti_3_C_2_T_x_ MXene, hybrid MIP–aptamer, steric signal-off

## Abstract

Pharmaceutical quality control faces an emerging problem with Tricyclic nitrosamine drug substance-related impurities (NDSRIs), for example, N-nitroso clozapine. The impurities preserve the compl ete structure of their parent active pharmaceutical ingredients, which consist of bulky lipophilic molecules with restricted conformational flexibility. The retention of bulky lipophilic structures from the parent active pharmaceutical ingredients in these impurities results in their complete overlap with other substances, which causes matrix ion suppression and weak ionization and unstable mass-spectrometric responses. The LC–MS/MS method functions as the official standard but its workflow requires excessive solvent use and produces complicated equipment systems, which prevent its use for routine ultra-trace monitoring. This narrative review analyzes hybrid electrochemical sensing technology to determine if it can solve the current problem by using tricyclic NDSRIs steric complexity as a functional benefit for detection purposes. The research combines data from nitrosamine detection with studies on porous 3D-printed electrodes, MXene electrochemistry, molecularly imprinted polymers, aptamer engineering and the computational systematic evolution of ligands by exponential enrichment (SELEX), molecularly imprinted polymer (MIP) and signal-off aptasensing. This review presents that planar electrodes create an unfavorable structure for bulky tricyclic analytes because they lead to surface obstruction and increased resistance during charge transfer. This review demonstrates that gyroid and TPMS-type porous scaffolds with a Ti_3_C_2_T_x_ MXene coating offer superior internal surface area, enhanced diffusion channels and accelerated electron movement. The dual MIP–aptamer system enables selective recognition by using MIP to perform shape-based target capture and specimen protection and aptamer to recognize specific molecular targets. The design process for aptamers includes E/Z nitrosamine isomer modeling, docking and molecular dynamics, and AI-based sequence selection methods, which form the core approach to targeting N-nitroso clozapine (N-CZN)-like proteins. This research outlines a new technique, which uses steric hindrance to block redox-probe entry, thus generating an electrical signal through current reduction. This review presents a fundamental operational model which supports the development of eco-friendly portable electrochemical devices to detect tricyclic NDSRIs with maximum sensitivity.

## 1. Introduction

The global regulatory crisis of 2018 emerged when N-nitrosodimethylamine (NDMA) was discovered in valsartan, which triggered a worldwide pharmaceutical quality control transformation [1]. Scientists quickly broadened their focus from basic nitrosamines to include nitrosamine drug substance-related impurities (NDSRIs) which develop when the active pharmaceutical ingredient (API) undergoes nitrosation during manufacturing and storage [2,3]. The analysis of NDSRIs, which form from tricyclic drugs including clozapine and olanzapine, becomes extremely difficult because of their complex structures and their strong affinity for lipophilic environments. The regulatory body uses liquid chromatography–tandem mass spectrometry (LC–MS/MS) to identify nitrosamines but this technique fails to perform well when tracking ultra-trace levels for everyday monitoring. The simultaneous occurrence of these impurities with the main active pharmaceutical ingredient (API) base substance at a concentration of one million times more than each other results in extreme matrix-based ion suppression, together with in-source fragmentation and matrix interference [4]. Standard chromatographic workflows depend on using significant amounts of organic solvents, which leads to poor green analytical chemistry performance (AGREE scores ~0.4), thus creating an immediate requirement for eco-friendly electrochemical systems which can be used in the field. The current performance gap, which requires regulatory acceptable-intake limits to reach sub-ppb levels, shows that there is an immediate need for advanced sensing technologies that provide high sensitivity and matrix tolerance and field operational capabilities.

Electrochemical sensors provide the exact set of features which are needed for this application. These devices use molecular recognition events on electrode surfaces to generate electrical signals, which they transform into measurable data for immediate detection without labels through compact equipment [5,6]. A standard electrochemical cell uses three electrodes, which consist of working, counter- and reference electrodes, while the working electrode receives recognition elements to identify the target substance [7]. The three transduction methods include amperometric, potentiometric and impedimetric systems, which provide different benefits when detecting particular analyte groups in various sample types [5,8]. For analytes with redox properties, direct electron transfer (DET) enables selective detection through its operation at the redox couple’s formal potential but mediated electron transfer (MET) relies on redox shuttles to diffuse because active sites become inaccessible due to steric or kinetic obstacles [9,10]. The detection of tricyclic NDSRIs depends on interfacial passivation because these compounds do not produce electroactive signals. The signal-off system works by having the large nitrosamine molecules attach to receptor-modified electrodes, which then block the movement of ferricyanide redox probes to create decreased electrical current signals [11,12]. The system functions according to its design but it responds strongly to changes in electrode construction and surface contamination.

Although planar electrodes require basic manufacturing operations their design fails to identify rigid tricyclic molecules of approximately 300–450 Da, which create passivation effects. The limited active surface area and two-dimensional design of their structure results in concentrated steric interference, which causes fast-rising interfacial charge-transfer resistance that produces poor signal stability [13,14]. The 3D porous structures of nanomaterial scaffolds and mesoporous films (2–50 nm) and 3D-printed electrodes solve these problems by establishing internal void areas that improve mass flow and enable redox probe movement and prevent total sensor contamination from large particle detection [11,15]. The review of (bio)sensor interfaces shows that hybrid polymer and nanomaterial thin film assembly methods enable exact surface chemistry and permeability management, which directly reduces fouling problems [16]. The design of mass transport control systems and surface passivation reduction methods now includes magnetic stimulation as a new approach to manage electrochemical interfaces [17]. The process of detecting NDSRIs in pharmaceutical matrices with multiple components needs elements which can separate NDSRIs from their structurally similar original medications. Scientists have developed a powerful new method which combines molecularly imprinted polymers (MIPs) with aptamers to create dual-recognition systems. The combination of MIPs with aptamers produces superior selectivity through their shape-selective cavities and their ability to bind with high affinity and specific conformations which also minimizes cross-reactivity [12,18,19,20]. The recent tutorial guidance about biosensor development states that the combination of receptors with proper electrode components will create dependable analytical devices that can move beyond laboratory testing [6].

There is a distinct gap between laboratory results and practical applications because electrochemical sensing has created advanced transduction systems and improved material development, yet scientists have not conducted enough validation experiments for tricyclic NDSRIs. A 2026 review presents a complete overview of sensor-based nitrosamine identification methods, which shows that most research focuses on detecting small nitrosamine molecules while scientists have yet to study the detection of bulky NDSRIs. The CRISPR/Cas12a-based electrochemiluminescence biosensor for NDMA detection on vertically ordered mesoporous silica films (2–50 nm) shows how advanced amplification techniques, together with nanostructured electrodes, produce high sensitivity but no similar systems exist for tricyclic NDSRIs [21]. The review establishes a new framework which links tricyclic NDSRI electrochemical detection mechanisms to real-world applications. Our research analyzes the primary obstacles, which include steric passivation and matrix interference and selectivity problems, by studying the combined operation of 3D electrode structures with MIP–aptamer dual-recognition systems and MXene-based conductive nanomaterials in a single detection system. The pharmaceutical quality control for clozapine must additionally resolve co-occurring tricyclic NDSRIs such as N-nitroso clozapine (N-CZN) and its related impurity N-CZNC, whose similar redox active aromatic core can generate overlapping steric signal-off responses. This review also focus on how such closely related analytes can be discriminated through differences in molecular volume and the degree of steric crowding at the electrode surface, through differences in receptor binding kinetics and the chemometric deconvolution of the composite electrochemical signal. This research study investigates artificial intelligence systems which could solve complex signal-off response problems while testing the manufacturing process scalability of additive manufacturing systems. This review establishes a regulatory framework which integrates basic electrochemistry principles with NDSRI-specific requirements to create precise electrochemical sensors for nitrosamine impurity detection.

## 2. Literature Search Strategy

This narrative review was developed through a structured literature search focused on analytical and sensor-design challenges relevant to bulky tricyclic nitrosamine drug substance-related impurities, with an emphasis on N-nitroso clozapine and related hybrid electrochemical detection concepts. The search covered major scientific databases, including Scopus, Web of Science, PubMed, and Google Scholar, using combinations of keywords such as ‘nitrosamine drug substance-related impurities’, ‘N-nitroso clozapine’, ‘tricyclic nitrosamines’, ‘LC-MS/MS nitrosamine analysis’, ‘molecularly imprinted polymer’, ‘aptamer’, ‘hybrid MIP-aptamer sensor’, ‘MXene electrochemistry’, ‘3D printed electrode’, ‘porous electrode’, ‘computational SELEX’, and ‘signal-off aptasensor’. The primary search focused on studies published from 2010 to 2025, with earlier seminal reports included when necessary to provide methodological or conceptual background. Records were screened for relevance to one or more of the following themes: (i) pharmaceutical nitrosamine detection, (ii) analytical limitations associated with bulky lipophilic NDSRIs, (iii) porous and MXene-based electrochemical transducers, (iv) MIP and aptamer recognition strategies, and (v) hybrid recognition systems applicable to trace impurity sensing. Articles were excluded when they were not directly relevant to nitrosamine analysis or electrochemical recognition, did not provide sufficient methodological detail, or were redundant with the inclusion of more comprehensive or recent reports. Priority was given to peer-reviewed studies with clear analytical scope and translational relevance to selective detection of structurally complex NDSRIs.

## 3. Electrochemical NDSRI Sensing: Progress and Limitations

### 3.1. Electrochemical Sensing: NDMA Progress, NDSRI Gap

Electrochemical sensing provides an effective option for nitrosamine analysis because it delivers fast results at minimal expense with portable equipment and minimal solvent usage. The research shows an obvious difference between the fast development of NDMA simple nitrosamine detection platforms and the complete lack of equivalent systems for tricyclic NDSRI detection.

Multiple electrochemical methods have proven to be effective for analyzing NDMA using their built-in analytical capabilities. The MIP-based sensors which use molecularly imprinted polymers achieve their best detection limits between parts-per-billion and femtomolar concentrations based on their interface construction and signal transmission technology. The polypyrrole-entrapped MIP/electrochemical impedance spectroscopy platform detected substances at microgram-per-liter concentrations while maintaining precise identification of drinking- and recycled-water contaminants [22]. The latest sensors achieved higher sensitivity levels through their design: [23] developed a reversible MIP-based electrochemical sensor which showed a detection limit of 1.16 ppb in water samples. The green-synthesized AgNPs/chitosan/3-APBA MIP electrode achieved femtomolar-level detection sensitivity through serum and tap-water samples while maintaining strong discrimination against related interferents [24]. The detection of NDMA and NDEA uses two different formats, which include a DNA carbon-dot biosensor and a CRISPR/Cas12a-based electrochemiluminescence system that detects NDMA at picogram-per-milliliter levels in complicated sample types [21,25]. Research studies have shown that MIP-based electrochemical sensors provide excellent selectivity through their compact design, which operates at low operating expenses, and they use environmentally friendly materials, yet their performance consistency and manufacturing scale and regulatory approval remain unresolved [26,27,28,29,30].

An electrochemical sensor which detects tricyclic NDSRIs including N-nitroso clozapine and its related rigid aromatic nitroso impurities remains undiscovered. The lack of such technology stands out because scientists have already created advanced electrochemical aptamer sensors and electrochemiluminescence detectors and impedance sensors and nanostructured biosensors which detect various small molecules, including adenosine, ATP, cocaine, tobramycin, doxorubicin, fumonisin B1, zearalenone, pesticides, toxins and metal ions [31,32,33,34,35,36,37,38,39,40,41,42]. The selection process for small-molecule aptamers remains difficult because these targets have limited binding areas and their binding interactions are difficult to predict [28,41,43]. The structural characteristics of tricyclic NDSRIs create barriers, which probably become more severe because their rigid aromatic structures and nitroso substitutions prevent them from achieving the conformational changes needed for structure-switching electrochemical aptamer sensor functionality [28,36,40]. The studies that examine aptamer-sensor stability through DNA nanostructures and xenonucleic acids and surface-engineering methods fail to identify aptamers which target aromatic nitrosamine impurities and tricyclic NDSRIs [44,45,46]. The development of electrochemical sensors for basic nitrosamines has reached a stage where these sensors can be used in field operations and quality control activities, yet no electrochemical platform based on MIPs, aptamers, ECL or CRISPR technology has been developed to detect tricyclic NDSRIs. The current research gap exists because recognition elements cannot detect tricyclic nitroso impurities which have complex structures, so scientists should focus on creating new sensors for this field.

### 3.2. Failure of Planar Electrodes

The rigid polycyclic structures and the bulky nitrosated amine groups, together with the weakly conductive aromatic systems of Tricyclic NDSRIs, create conditions which block interfaces instead of supporting efficient electron transfer at electrode surfaces.

Research has not yet produced direct electrochemical measurements for tricyclic NDSRIs but multiple studies using electrochemical impedance spectroscopy (EIS) and self-assembled monolayers and aromatic amine oxidation and porous-electrode analysis have established a unified mechanistic framework to understand these compounds.

The main concept involves surface-coverage-dependent blocking which uses θ to show how much electrode surface area becomes blocked by an adsorbed layer or an insulating layer. The increasing value of θ reduces the available conductive surface which ferri/ferrocyanide and similar probes can access, thus leading to higher charge-transfer resistance (Rct) and an expanded Nyquist semicircle [47,48,49,50]. The tricyclic aromatic molecule which faces upward functions as a protective “molecular umbrella”, which shields the surface from both probe molecules and target molecules [47,48,49]. Aromatic adsorbates including dopamine support this interpretation because their oxidation products create a protective layer on carbon electrodes, which leads to higher Rct values. The formation of thicker organic and biomolecular interfacial films results in decreased electron transfer efficiency because these films block electron movement [50,51,52].

The amine center exists in this situation because of steric influences, which establish this particular challenge. The electrooxidative grafting process enabled primary amines to obtain a surface coverage three times greater than what secondary amines achieved but tertiary amines failed to immobilize because of their nitrogen atom steric blockage [53]. The ferricyanide transfer process faced obstacles because of the bulky amino-β-cyclodextrin layers, which also resulted in wider voltametric peak shapes [54]. The nitrosated nitrogen in tricyclic NDSRIs gets embedded into a large and fixed aromatic structure, which likely prevents efficient surface contact and results in compact molecular arrangements when the molecules settle [53,54].

The present electronic components generate multiple performance problems which result in performance degradation. The oxidation process of aromatic amines takes place through multiple stages, resulting in permanent chemical changes. The nitrosation process will draw electrons from nitrogen, which will disrupt the π-system so the nitrosated compound will show reduced electrochemical activity compared to its original amine form [52,55,56,57,58]. The electrical conductivity of aromatic amine-based electrochemical polymers reaches extremely low levels because they exhibit conductivities of about 5 × 10^−13^ Ω^−1^·cm^−1^.

Research on porous electrodes demonstrates that three-dimensional structures operate more effectively than other available designs. The 3D porous structures maintain their distributed active sites, expanded surface area and multiple diffusion channels, which enable them to handle partial blockages and elevated local θ values better than flat electrodes [59,60,61]. The research shows that tricyclic NDSRIs will block planar electrodes but 3D or porous electrodes provide the best solution for blocking steric exclusion and high Rct and nitrosation-perturbed electron transfer problems.

## 4. 3D-Printed and MXene-Based Transduction Platforms

### 4.1. Porous 3D Electrodes for Tricyclic NDSRIs

Conventional electrodes cannot handle bulky tricyclic NDSRIs because their shape is the primary restriction instead of their electrical conductivity. The external 2D surface of flat glassy carbon electrodes and screen-printed electrodes and most fused-deposition-modeling (FDM) carbon/polymer electrodes allows for large rigid analytes to form blocking molecular umbrella layers through their restricted contact area. The current body of research about additively manufactured electrochemical sensors shows that FDM-printed graphene/PLA and carbon-black/PLA electrodes perform similarly to standard planar carbon electrodes until users conduct multiple activation procedures to reveal their conductive filler and decrease their electrical resistance [62,63,64,65,66,67,68]. FDM alone does not overcome steric exclusion.

The main benefit of additive manufacturing becomes apparent when this process produces three-dimensional porous structures which span entire volumes. The research fields of analytical science and catalytic science and energy device development value 3D-printed lattices, TPMS structures and gyroid scaffolds because these materials enable separate control of porosity and pore dimensions and flow path complexity, which creates extensive internal surface area from minimal external dimensions [65,69,70,71,72,73,74]. The TPMS Gyroid structure type has gained importance because it forms a bicontinuous system which provides complete network connectivity for better fluid movement through its lower tortuosity than both packed beds and irregular foam structures [71,75,76]. The structure maintains its physical form with an 80% porosity level, which creates the necessary open framework to handle aromatic impurities that face steric restrictions [65,73].

Technology choice therefore matters. The FDM/FFF process creates features larger than 100 µm but users must perform sanding, solvent or alkaline treatment, electrochemical activation and laser scribing to achieve the desired results. The SLA/mSLA process produces better TPMS lattice precision through its higher resolution but users must complete post-curing and resin-removal operations after printing. The selective laser melting process creates strong metal TPMS electrodes but its high price makes it unsuitable for sensing applications [62,63,64,65,66,67,68,70,73,77,78].

Research on similar electrochemical porous systems has shown that this mechanism produces superior results. The architected porous electrodes provide better electrochemical active surface area and lower transport resistance and charge-transfer impedance than their flat counterparts [15,79,80,81,82,83]. The DLP-printed porous SnO2 electrode demonstrated a charge-transfer impedance twenty-five times lower than what thick planar electrodes exhibited [84]. The 3D electrodes which were optimized for flow conditions demonstrated enhanced mass transfer capabilities when operating under transport-limited conditions [83,85]. Structured printed surfaces have been used to analyze rigid tricyclic molecules of approximately 300–450 Da, which include atorvastatin, tetracycline, steroid hormones and nucleobases. The research on hierarchical porous platforms which use mesoporous electrodes (2–50 nm) and MXene-based hierarchical porous structures comprising macropores (>50 nm), mesopores (2–50 nm), and micropores (<2 nm) shows that multiscale porosity enhances the adsorption process of organic molecules through better accessibility and improved kinetics [74,75,86,87,88,89,90,91,92].

Direct studies on tricyclic NDSRIs are still absent, but the converging evidence is clear: planar and activated FDM electrodes remain surface-limited, whereas 3D porous and TPMS architectures provide higher internal area, lower tortuosity, more diffusion pathways, and less vulnerability to local blocking. The electrochemical detection of rigid ~400 Da tricyclic NDSRIs depends on these fundamental characteristics, which make them suitable for measurement.

### 4.2. Conductive Filaments and Nanocomposite Integration

The 3D structure of tricyclic NDSRI sensing requires additional factors to function because its performance depends on the composition of conductive filaments and the activation process after printing and the integration of molecularly imprinted polymers (MIP). Research has established two separate but fully developed fields, which include MIP-based electrochemical sensors that deliver precise detection at minimal concentration levels and PLA/carbon-based printed electrodes which show improved charge-transfer resistance (Rct) through proper activation procedures. A single 3D-printed platform which integrates all nitrosamine detection systems remains to be developed.

The scientific community has established MIPs as electrochemical recognition layers which function effectively. The scientific community has developed electropolymerized MIP films through recent studies which use conducting or semiconducting matrices including polypyrrole and PEDOT and combine nanoMIPs with magnetic MIPs and hybrid MIP/nanomaterial systems to achieve detection limits between sub-nanomolar and femtomolar for drugs, biomarkers, pesticides and ions [26,93,94,95,96,97]. The selectivity of the system depends on the shape of the cavities and the selection of functional monomers and the ability to rebind targets effectively instead of requiring an additional activation process [30,98,99]. The current literature reviews exclude nitrosamines from their content while scientists have yet to develop an electrochemical MIP sensor which detects tricyclic NDSRIs. The scientific community has only identified NDMA through a traditional non-printed electrode which uses MIP particles that were pre-made and combined with a polypyrrole matrix instead of using a 3D-printed substrate [94].

At the same time, the materials literature shows that PLA-based conductive composites can be transformed from mediocre printed conductors into useful electrochemical transducers. FDM electrodes made from PLA/carbon black and PLA/graphite materials need surface treatment because their PLA surface layer prevents the conductive filler network from being visible [100,101]. The surface PLA layer removal through NaOH or DMF chemical activation, electrochemical polarization at 1.8–2.5 V and atmospheric air plasma treatment leads to carbon domain exposure, porosity enhancement and better surface wettability, which results in reduced Rct values and faster heterogeneous electron transfer [100,102,103,104]. The activation process enabled 28–30 wt% carbon-black PLA filaments to detect catechol and hydroquinone but PLA/graphite filaments with 40 wt% graphite content became functional sensors for uric acid, dopamine, metal ions, and SARS-CoV-2 protein detection [101,105]. The research shows that coconut-shell biochar works as a sustainable filler because it lowers Rct values from 9.1 kΩ to 1.0 kΩ in conductive PLA and it enables the detection of carbendazim at concentrations as low as 0.01 µM [106].

The critical gap, therefore, is not whether MIPs work or whether activated conductive filaments work; both do. The existing research lacks studies which combine nitrosamine-specific MIPs with 3D-printed conductive substrates for studying bulky tricyclic NDSRIs. The current MIP electrochemical sensors operate with tamoxifen, axitinib, vismodegib, citalopram and amoxicillin, which proves that molecular imprinting technology functions effectively, with analytes weighing between 324 and 421 Da [107,108,109,110,111]. The development of a new platform should combine a PLA/carbon or PLA/biochar scaffold which operates sustainably with an electropolymerized or nanoparticle-based MIP layer that enables shape-selective recognition to solve the existing gap in nitrosamine electroanalysis.

### 4.3. MXenes for Aptamer-Compatible Transduction

Ti_3_C_2_T_x_ MXene functions as an effective signal booster for tricyclic NDSRI detection because it delivers rapid heterogeneous electron-transfer (HET) reactions on its hydrophilic surface, which provide better compatibility with aptamers than graphene-based materials. The latest mechanistic study demonstrated that ferri/ferrocyanide reactions proceed with a standard rate constant of 0.03 cm s^−1^, which exceeds V_2_CT_x_ by about five times because of its elevated density of states and quantum capacitance [112]. Research reviews reveal that Ti_3_C_2_T_x_ functions as an effective charge-transfer mediator, which operates as an electrocatalytically active transducer that delivers electroanalysis results which surpass graphene-based systems [113,114,115].

The sensor delivers superior performance because of this advantage. The MXene-based electrochemical platforms achieve detection limits which range from low-nanomolar to picomolar levels for drugs, toxins, pesticides and biomarkers [114,115,116,117,118,119,120,121]. The Ti_3_C_2_T_x_-AuNP aptasensor for chloramphenicol demonstrated superior performance through reduced peak separation and lower charge-transfer resistance when compared to bare GCE, which resulted in an LOD of 0.03 pM [119]. The Ti_3_C_2_T_x_ heterostructures presented in research have demonstrated their ability to detect ciprofloxacin, nitrofurantoin, benomyl and methylmalonic acid at concentrations which range from sub-nanomolar to picomolar levels [116,119,121,122].

Surface chemistry serves as the second primary benefit of this system. Ti_3_C_2_T_x_ contains multiple surface terminations which include –O, –OH and –F groups that make it hydrophilic and enable different biological attachment methods [123,124,125]. The groups enable mild methods for aptamer attachment through phosphate–Ti bonds, the EDC/NHS method and thiol-based attachment using AuNP or polydopamine layers [114,116,117,126,127]. The method proves essential for bulky tricyclic NDSRIs because their binding with aptamers requires maintaining complex protein folding instead of achieving optimal probe numbers.

Available studies support the stability of MXene–biomolecule interfaces. Ti_3_C_2_T_x_-based platforms have been reported for saxitoxin, exosomes, tetracycline, osteopontin, and cancer-related targets, often with good selectivity and operational stability [117,126,128,129]. The Ti_3_C_2_T_x_/PMo_12_/polypyrrole nanohybrid system allowed for the permanent attachment of osteopontin aptamers [115] while the aptamer-modified Ti_3_C_2_ nanosheets demonstrated their ability to function within biological systems [130]. The process of oxidation creates an actual obstacle because it transforms surfaces, which results in lower conductivity and unpredictable results, so scientists need to develop stabilization methods which include polymer encapsulation, hydrogels and metal- or MOF-based heterointerfaces [114,116,123,124,128,129,131]. Figure 1 summarizes the factors governing advanced electrochemical transducers.

The research field contains a vital missing element because scientists have not created MXene-based aptasensors, which detect N-nitrosamines or tricyclic NDSRIs [113,114,129,132]. The Ti_3_C_2_T_x_ material demonstrates suitability for detecting tricyclic NDSRI through its rapid HET response, minimal Rct values, extensive active surface area and water-attracting surface, which supports aptamer binding.

### 4.4. MXene–3D Integration: Progress and Gaps

Scientists utilize MXenes with 3D-printed electrodes to develop advanced electrochemical sensors but three fundamental technical obstacles prevent their full operation. The three fundamental technical obstacles which control system performance include nanosheet restacking suppression, oxidation-driven degradation reduction and the process of converting planar MXene interface sensitivity into printed three-dimensional structures. The literature shows that restacking functions as the main structural problem because Ti_3_C_2_T_x_ and other MXene sheets experience van der Waals forces which decrease their available surface area, their ability to conduct ions and their active-site visibility after they have been deposited or printed.

Multiple methods exist that can solve this problem. The areal capacitance of activated 3D carbon electrodes improved through MXene post-print coating, which a single study demonstrated by showing a 3× increase from 45 to 135 mF cm^−2^ after electrochemical activation, while keeping the original shape and achieving 92% capacitance stability throughout 5000 cycling tests [133]. The engineering of ink materials through direct ink writing and inkjet methods prevents restacking by creating crumpled N-doped MXene inks, which form corrugated flakes that expand their interlayer distance from 1.35 to 1.62 nm and achieve an aerial capacitance of 8.2 F cm^−2^ [134]. Spacer-mediated composites enable better access to their components; the insertion of graphene material into the structure expanded the gallery spacing to 1.85 nm, which resulted in a volumetric capacitance increase from 380 to 562 F cm^−3^ [132].

Three-dimensional porous architectures serve as an alternative method to maintain electrochemical accessibility. The Breath-figure-assembled porous MXene films developed surfaces with larger areas than their dense counterparts, which allowed them to detect dopamine at concentrations starting from 0.17 μM. The zinc-templated MXene/graphene macroporous (>50 nm) frameworks maintained 94% of their capacitance after 20,000 cycles while showing excellent performance at high current levels [15]. The structures derived from aerogels and hydrogels create low-density networks which provide excellent conductive paths for wearable and flexible electronic devices [135,136].

The second main limitation of oxidation occurs when aqueous processing and storage at room temperature take place. The MXene material in its original state experiences fast declines in electrical conductivity when it comes into contact with air or water, which leads to TiO_2_ oxidation that takes multiple days to weeks to develop depending on how it is stored [137,138]. Practical stabilization strategies are therefore critical. The electrical conductivity of sodium ascorbate capped materials stayed at 92% after 80 days of ambient exposure while the method kept aqueous inks stable [139]. The combination of conformal polydopamine coatings with polymer-confined hydrogels lowered oxidation rates because they prevented H_2_O/O_2_ from attacking interfaces and from spreading through the material [138,140]. The carbon-hybrid structures perform best because their MXene/graphene 3D networks maintain their conductivity levels after 60 days of air exposure [140].

A third and less resolved issue is analytical sensitivity. The biosensors made from planar MXene materials detect biomarkers at femtomolar to picogram levels but 3D-printed MXene electrodes only demonstrate nanomolar to micromolar detection limits for dopamine, H_2_O_2_, glucose and uric acid [138,141]. The research identifies a gap in translating the scientific findings to practical applications rather than any fundamental restrictions in the materials. The research indicates that printed MXene structures should enable picomolar detection because they provide extensive electroactive surfaces and strong interfacial capacitance and work well with amplification chemistries. The literature suggests picomolar detection will become possible through the establishment of a unified system which combines optimized receptor functionalization with low-noise transduction and extended stability. The primary obstacle to tricyclic NDSRIs using MXene–3D integration is the gap between the feasibility of its implementation and its ability to achieve ultra-trace selectivity when testing pharmaceutical substances. Table 1 presents the quantitative results, which stem from 3D printing and conductive composite systems and MXene-enabled systems, while showing the performance features which matter most for bulky tricyclic NDSRIs. Figure 2 shows the development from geometric transducer design to molecular-recognition engineering through a visual representation which demonstrates the transformation of tricyclic NDSRIs steric interference into an electrochemical detection system by using porous 3D–MXene structures and optimized aptamer interfaces through computational methods.

## 5. Aptamer Engineering for Tricyclic NDSRIs

### 5.1. Specificity Challenges in Tricyclic NDSRI Detection and Limits of Empirical SELEX

Tricyclic NDSRIs such as N-nitroso clozapine (N-CZN) rarely occur in isolation; they can coexist with other nitrosamine impurities arising from clozapine and its related substances, such as N-nitroso clozapine impurity C (N-CZNC), creating a composite-signal problem where multiple near-identical species generate overlapping electrochemical responses. While three-dimensional porous electrodes improve mass transport and alleviate steric blocking, they cannot provide the molecular-level discrimination required to distinguish one tricyclic nitrosamine impurity from another. This specificity challenge is therefore recognition-driven. Electrochemical aptamer sensors, despite their programmability, often lack sufficient selectivity against chemically similar interferents, though surface-charge tuning has been shown to discriminate molecules differing by a single hydroxyl group and to quantify mixtures using multi-electrode arrays [157]. In contrast, molecularly imprinted polymer (MIP)-based sensors have demonstrated exceptional selectivity for nitrosamines: an NDMA-imprinted sensor achieved a limit of detection (LOD) of 3.63 fM and remained selective in the presence of NDEA, sartans, and common biological interferents [24], while a complementary NDEA-imprinted sensor reached 5.92 fM LOD in standard solution and retained selectivity against NDMA and serum components [158]. Further enhancing performance, a magnetic Ti_3_C_2_T_x_/Fe_3_O_4_ MIP sensor exploiting MXene’s fast electron transfer attained an LOD of 2.33 × 10^−16^ mol L^−1^ for methylmalonic acid [159], illustrating how MXene–MIP hybrids combine high conductivity with precise molecular recognition. A dual sensor based on PtNPs/MWCNTs/Fe-porphyrinic MOF achieved simultaneous detection of NDPhA and NDEA with LODs of 6.53 × 10^−3^ μg mL^−1^ and 0.173 μg mL^−1^ and retained 92.6% of its current after 30 days [160]. These findings establish a clear division of labor: 3D architectures solve transport and accessibility, while aptamer, MIP, or MIP–Aptamer dual recognition provide the essential specificity needed to resolve closely related tricyclic NDSRIs in pharmaceutical matrices.

The process of selecting aptamers for tricyclic NDSRIs through standard empirical SELEX methods encounters significant challenges because the fundamental principles of enrichment and partitioning and target presentation break down when working with small rigid hydrophobic analytes. The latest SELEX reviews have identified three major obstacles, which consist of immobilization artifacts, insufficient target chemistry information and ineffective separation methods for small molecules with weak charges. The combination of these elements makes an exclusively experimental approach inefficient when working with tricyclic NDSRIs, which supports the use of computational selection methods and combined selection approaches.

The first problem is target immobilization. Traditional bead-based SELEX requires that researchers attach analytes permanently to Sepharose beads, magnetic beads and similar matrix materials. Research studies have shown that this method fails to identify free targets because it detects target–matrix complexes which modify the natural structure of the target and its binding regions [161,162,163,164]. The process of adding linkers creates major difficulties for rigid tricyclic NDSRI because it conceals the nitroso-bearing amine and it forces the aromatic structure to adopt an unnatural position. The development of Capture-SELEX as an immobilization-free format solved some problems but it still uses indirect methods because the capture chemistry operations move to the oligonucleotide domain [163,164,165].

Small molecules have an inherent chemical limitation which forms the basis of the second problem. The process of small-molecule SELEX demonstrates poor effectiveness because substances under 1000 Da lack sufficient functional groups, which restricts their ability to bind with nucleic acids [166,167,168,169]. The actual success percentage remains unknown because studies about unsuccessful selections do not reach publication, thus creating a publication bias which affects hydrophobic targets in particular [163,164]. The Capture-SELEX method faces three main challenges, which include producing false-positive results, creating weak target binding and losing its pool of diverse candidates over time [164,169].

Partition-based formats including CE-SELEX face a third operational restriction. The method avoids target immobilization through its use of electrophoretic mobility to identify free DNA from DNA–target complex patterns. Small molecules experience minimal changes in their mobility, which results in poor enrichment performance [170,171,172]. The situation will probably become worse for tricyclic NDSRIs because their DNA binding produces only small changes in hydrodynamic and charge characteristics.

Research indicates that scientists can achieve optimized SELEX for small molecules through purposeful engineering instead of random selection methods. The use of biased libraries enhances the chances of finding hits while nuclease-assisted selection methods lead to faster results and better affinity results [169,173]. The Capture-SELEX method requires between 8 and 20 selection cycles for DNA and between 6 and 12 selection cycles for RNA yet robotic systems generate only micromolar-affinity aptamers which need further optimization [164,174]. The best approach for tricyclic NDSRIs involves using a combined method which includes computational pre-screening, biased library design, optimized small-molecule selection formats and post-selection refinement [175].

### 5.2. Computational Aptamer Design

Scientists now use in silico aptamer design to create a predictive discovery process which builds on the traditional SELEX method. Scientists need this method because they do not understand small molecules, even though standard selection methods fail to work because of poor enrichment and high experimental failure rates. Scientists now agree on three main research methods, which include molecular dynamics (MD) with free-energy analysis, deep learning on SELEX sequencing data and iterative in silico affinity maturation [176,177,178]. The structural foundation for computational aptamer engineering now depends on MD-based workflows. The typical pipelines start by filtering secondary structures through RNAfold and Mfold before they proceed to 3D modeling and docking, followed by explicit-solvent MD and MM-PBSA or MM-GBSA refinement to find stable complexes with low ΔG values [176,178,179,180]. Scientists have discovered that the structure with the lowest energy in a static state does not represent the actual binding configuration. The use of multiple MD simulations to generate metastable-state ensembles led to better docking results for AMP and L-argininamide with aptamers. Scientists observed multiple conformations of tetracycline-class analytes and chloramphenicol which demonstrated that single-structure docking would produce incorrect ranking results [181,182,183].

Research using quantitative methods demonstrates that MD-derived energetics enable successful predictions. The F20 aptamer demonstrated the best MM-PBSA binding free energy in the aflatoxin B1 research, which matched the experimental affinity results from [184]. The selection process for dopamine- and safranal-binding aptamers used docking energy, RMSD, RMSF and radius of gyration measurements. The MD plus MM/PBSA method demonstrated its ability to track experimental Kd values from different Alzheimer’s-related aptamer classes [185,186,187]. The research area employs GROMACS-based MD with MM-PBSA as its primary computational framework [176,178,184,188].

The second foundational element consists of applying deep learning techniques to analyze SELEX sequencing results. DeepAptamer achieves better predictive results by combining sequence data with structural information through its hybrid convolutional neural network and bidirectional long short-term memory model, which also identifies high-affinity sequences from early weakly enriched rounds [181,182,189,190,191]. The AUROC values of other AI models reached 0.998 and their classification accuracies reached 0.995, yet AptaTrans continues to advance the field by developing methods for interaction prediction and generative optimization [192,193,194].

The third pillar is in silico affinity maturation, where docking, MD, hotspot mapping, and mutation or truncation are iteratively applied until improved ΔG or stability is achieved [176,177,178]. The genetic-algorithm-guided maturation process for AFB1 produced variant improvements which matched the actual experimental results and the APTAMD method allowed researchers to eliminate 20–35% of amino acids without decreasing binding capability [179,184]. These studies prove that computational design has evolved past its initial role as a supplementary tool. The tricyclic NDSRI workflow requires secondary-structure filtering to create multi-conformer 3D models which undergo isomer-aware docking and MD-based free-energy refinement before deep-learning-based prioritization and multiple rounds of truncation and mutation for experimental testing. Secondary-structure screening, three-dimensional ensemble generation, molecular docking, and molecular-dynamics refinement collectively define the conformational states and potential binding pockets of candidate aptamers, as conceptually summarized in Figure 2.

### 5.3. N-CZN Aptamer Design Rules

The development of N-nitroso clozapine (N-CZN) aptamers needs a specific process which handles nitrosamine stereochemistry, aromatic recognition and molecular flexibility instead of treating the target as one fixed ligand. The research community has not yet conducted any studies about N-CZN but scientists use asymmetric N-nitrosamines and DNA aptamers with known structures to develop their target preparation and docking and validation methods.

The initial requirement needs explicit procedures to handle E/Z isomerism. The research on asymmetric N-nitrosamines through DFT and variable-temperature NMR experiments revealed different configurational populations which showed Z/E shifts between 10% and 60% and conformer-specific stabilization variations of approximately 5 kcal/mol, which established that N–N/N–NO rotation was limited and required separate modeling for each nitrosamine isomer [189,195]. The research from previous conformational studies demonstrated that nitrosamine systems and hindered cyclic amines possessed limited rotation abilities and multiple possible structural arrangements, which supported the need for dedicated conformer creation instead of using single-structure docking [190,191,196].The research demonstrated through computer modeling that nitrosamine stability, hydrogen bond patterns and adsorption strength varied according to molecular configuration, which created distinct binding strength variations between E and Z molecular structures [197,198]. The research on stereochemically sensitive ligands through consensus docking studies demonstrated that E and Z forms produced different ranking results which showed various levels of agreement with experimental data; thus, researchers should perform docking and apply weighted scoring separately for each isomeric form [199].

Once the target is prepared properly, the most plausible binding motif for N-CZN is a mixed interface dominated by π–π stacking and reinforced by hydrogen bonding. High-resolution aptamer structures strongly support this model. The planar polyaromatic ligand of the aflatoxin B1 aptamer exists inside a DNA pocket, which achieves stability through its interaction with guanine-rich sequences in the surrounding region [200]. The ochratoxin A aptamer uses a duplex–quadruplex junction to create ligand affinity through π-stacking, hydrophobic interactions and halogen bonds, which result in a 100-fold increase in binding strength compared to ochratoxin B [201]. ATP-binding DNA aptamers offer matching information through MD and energy decomposition which reveal that dispersion and π-stacking interactions produce stronger stabilization than any individual hydrogen bond, yet hydrogen-bonding residues continue to determine specificity [202,203]. Research on aptamer–small-molecule interfaces has established a common pattern which shows that π-stacking, hydrogen bonding, hydrophobic packing and functional-group complementarity serve as the main recognition elements for small molecules [204,205,206]. The design of functional groups for 27 small-molecule targets proves that scientists must define aromatic groups and donor/acceptor groups during sequence selection and ranking [207]. The structure of N-CZN exhibits a three-ring aromatic core which forms a stacked sandwich arrangement that ends with a cap structure, while the nitroso oxygen forms multiple hydrogen bonds through direct contact or through water molecules.

The virtual-screening workflow operates based on these structural precedents, which establish its foundation. The process of secondary structure creation begins with Mfold-like tools before scientists build multiple three-dimensional ssDNA structures from each sequence to identify the different pocket formations which result from alternative folding patterns. Another method applied RNA Composer to analyze three different steady-state tertiary structures from each sequence during their tetracycline aptamer investigation, yet the strongest binder did not match the structure which had the lowest overall energy [183]. The computational aptamer design process needs 2D filtering and 3D ensemble generation and docking and refinement to function as a continuous workflow instead of operating as a single-model system [177,204]. For N-CZN, the docking process requires both E and Z isomers to undergo blind docking against all aptamer conformations using two separate docking programs before performing energy-based selection and consensus pose determination through cluster analysis. The final selection of candidates needs to undergo validation through alternative Kd measurement methods and testing with mutants which disrupt binding motifs because small-molecule aptamer Kd values show differences between measured and actual thermodynamic Kd values depending on the testing method and the time needed for system equilibrium [199,205,206]. Representative small-molecule aptamer complexes indicate that aromatic targets are commonly stabilized through π–π stacking and hydrophobic packing, whereas hydrogen bonding and electrostatic complementarity provide additional functional-group specificity, as shown in Figure 2. The research community supports a structured approach to N-CZN aptamer development which includes separate analysis of E and Z forms, prediction of stacking-based motifs, multiple docking simulations and detailed evaluation of the docking results. Table 2 presents the numerical limits which control small-molecule SELEX operations while displaying the digital tools that operate on N-CZN-like targets.

### 5.4. Steric-Hindrance Signal-Off Aptasensing

The basic operation of signal-off electrochemical aptasensors depends on target binding, which forms a physical shield that blocks electron flow and reporter contact to generate measurable decreases in current and higher impedance levels. The tricyclic NDSRIs with rigid tricyclic molecules of approximately 300–450 Da show strong responses to this method because their size creates a significant signal-suppression effect which scientists usually find difficult to analyze. Translation of these molecular-recognition events into an analytical response further requires controlled aptamer immobilization and an electrode configuration capable of converting target-induced folding or steric blocking into a measurable signal decrease, as illustrated in Figure 3.

The most widely demonstrated implementation uses solution-phase ferri/ferrocyanide as the redox probe. The formation of target molecules on the electrode surface creates two types of physical and electrical obstacles which produce rising Rct values according to the reusable impedimetric aptasensor research by Radi and colleagues in 2005. The binding of chloramphenicol to aptamers created a structural change which prevented [Fe(CN)_6_]^3−^/^4−^ from entering because of physical blocking and electrical opposition [209]. The research by Reich and colleagues in 2017 demonstrated that Staphylococcus aureus Protein A aptasensors showed the same Rct increase, which matched the target quantity. The same pattern appeared in the graphene aptasensor for Acinetobacter baumannii detection which used no labels because the cell attachment to surfaces became the major factor in measurement outcomes [210]. Signal suppression produces quantifiable effects which impact system performance. The square-wave voltammetry method revealed that heme-based target binding reduced current flow by 24–31% when concentrations reached micromolar levels [211]. The SARS-CoV-2 aptasensors, which depend on folding methods, achieved a detection limit of 7 nM through S1 binding, which caused steric interference that moved their attached redox marker [212]. The steric-hindrance aptamer assay that blocks complementary strand hybridization detection achieved SARS-CoV-2 pseudovirus detection limits of 89 copies/mL in buffer and 1505 copies/mL in saliva for larger targets while achieving perfect diagnostic performance with 20 clinical samples [213]. The Omicron-specific detection system achieved a detection limit of 247 copies/mL in buffer solutions [213].

Nanostructured transducers generate an increased output, which produces a stronger effect. The researchers achieved detection limits of 1 pM for acetamiprid and atrazine by using Platinum nanoparticle microwires which blocked electron transfer through aptamer-based steric interference [214]. The ratiometric photoelectrochemical/electrochemical streptomycin sensor demonstrated that surface accumulation controls both detection methods equally [215]. The ferrocene/cDNA-MXene probe serves as a competitive MUC1 aptasensor, which reaches an LOD of 0.33 pM through its ability to detect target molecules by monitoring changes in current caused by probe detachment [216]. The evidence demonstrates that researchers should reverse their current design approach for tricyclic NDSRIs on nanoporous MXene because the same steric bulk which stops chromatographic and planar-electrode analysis now functions as the main detection mechanism when recognition occurs at a small area with a high surface area where MXene nanochannels serve as molecular stoppers. Table 3 presents evidence-based analytical methods which use signal-off aptasensing platforms to show how researchers use steric hindrance for bulky target electrochemical sensing instead of dealing with it as a problem.

## 6. AI-Assisted Signal Deconvolution

### 6.1. The Composite-Signal Problem

Quality control for clozapine must identify multiple nitrosamine impurities instead of relying on a single one. The latest LC–MS/MS research found N-nitroso clozapine (N-CZN) occurs with N-nitroso clozapine impurity C (N-CZNC) which requires the detection of both substances together instead of separate tests. The validated green LC–MS/MS method achieved perfect linearity (R^2^ 0.999 from LOQ to 150%) during the quantification of both substances in clozapine drug substance and its pharmaceutical formulations. The detection limits for N-CZN and N-CZNC differed because N-CZN had LOD/LOQ values of 0.058/0.177 µg mL^−1^ and N-CZNC had LOD/LOQ values of 0.014/0.044 µg mL^−1^. The recovery rates for the method fell between 88% and 102% [223]. The research identifies a natural connection between clozapine and its related substances, which mirrors current pharmaceutical regulations that demand simultaneous control of various drug-specific nitrosamines including those found in ciprofloxacin, moxifloxacin, clonidine, trimetazidine, duloxetine, citalopram, and sitagliptin impurities [224,225,226,227,228,229,230,231].

For electrochemical sensing, this creates more of a challenge than simple trace detection: mixture discrimination. Although N-CZN and N-CZNC are structurally related tricyclic nitrosamines with similar aromatic frameworks, they are chemically distinct species with different molecular weights and distinct LC–MS/MS transitions and their similar redox-active aromatic cores may produce overlapping voltametric features, necessitating mathematical separation for accurate quantification. The electroanalytical literature shows that such systems routinely collapse into a single unresolved voltametric feature unless mathematical separation is applied. Another study showed that Kalman filtering enables the separation of voltametric signals that overlap when their components exist at different concentrations of about 2.5 mV, yet the original peaks become indistinguishable [232]. Multiple studies have observed the same pattern of behavior in different chemical systems which share common analytical characteristics. A similar study used Gaussian deconvolution to separate the combined signals which square-wave voltammetry produced from dissociated and undissociated species in acid mixtures [233]. The optimized conditions for α-, γ-, and δ-tocopherol did not achieve complete separation of the anodic waves, which required signal deconvolution for simultaneous determination. The detection limits for these compounds range from 0.89 to 3.72 × 10^−6^ mol L^−1^ and their recoveries span from 72% to 128.5% [234]. The oxidation of levamisole at +0.85 V in seized cocaine samples produced strong interference with cocaine oxidation at +0.96 V, which required signal processing after data collection to achieve accurate identification. The deconvoluted electrochemical data showed strong agreement with standard analytical results through a correlation coefficient of r = 0.995 [235,236]. The presented examples indicate that N-CZN and N-CZNC will produce a merged peak instead of separate volt.

The practical implication is that electrochemical multi-NDSRI sensing must move beyond peak-height calibration. The combination of FFT square-wave voltammetry with partial least squares analysis enabled the detection of three dihydroxybenzene isomers through RMSEV values between 0.042 and 0.066 µM and LODs at 0.045 to 0.046 µM [237]. The direct orthogonal signal correction method together with wavelet packet transform and PLS delivered better results than basic chemometric models for analyzing nitroaniline isomers, which showed extreme peak overlapping [238]. The latest square-wave-based analytical methods, which include potential-corrected multi-sampled cumulative square-wave voltammetry and Faradaic spectroscopy, produce improved data sets for analysis [239]. The current-averaging technique enables partial differentiation between analyte signals and interfering. The research articles indicate that clozapine quality control faces a complex analytical problem because it needs both specific detection methods and mandatory signal separation through deconvolution or chemometric techniques. For electrochemical platforms, structurally similar NDSRIs are unlikely to be resolved by raw voltammetry alone. The most realistic strategy therefore combines selective recognition, optimized square-wave methods, and compulsory deconvolution or chemometric separation of composite signals. The signal-off system serves as the initial stage of the process because the binding reaction generates a combined electrochemical signal from N-CZN/N-CZNC recognition, which requires machine learning to separate its components. Table 4 summarizes the key experimental evidence defining the multi-NDSRI mixture dilemma for clozapine electrochemical quality control.

#### Mechanistic Basis for N-CZN/N-CZNC Discrimination

N-nitroso clozapine (N-CZN) and N-nitroso clozapine impurity C (N-CZNC) both generate signal-off electrochemical responses due to steric hindrance at the nanostructured transducer interface, which occurs due to the bulky tricyclic structures of both analytes [18,164]. Since both analytes give rise to similar current suppressions, identification of the analyte is not possible from the electrochemical response alone [47,50]. Separation of the analytes can be achieved through three mechanisms. The first difference is the molecular volumes of the analytes, which result in different degrees of steric crowding at the electrode surface [47,50]. The second is that the binding kinetics between the analytes and their receptors are different and can be determined by analyzing the transient signal-decay profiles of each analyte [164,199]. The third mechanism is the chemometric techniques that can be applied to the electrochemical data to resolve the analytes, including techniques like Kalman filtering [232], Gaussian deconvolution [233], and partial least squares regression [241]. The responses of the analytes must be anchored to an orthogonal reference standard to enable the chemometric deconvolution of the electrochemical data [223]. The study also demonstrated an LC–MS/MS method for the determination of N-CZN and N-CZNC, with detection limits of 0.058 and 0.014 ug/mL, respectively. Using this validated method, the electrochemical data can be mapped to the LC–MS/MS outputs that allow for the construction of multivariate models, which can distinguish the analytes based on their mass and retention-time fingerprints. Thus, machine learning approaches to data analysis do not pertain to the analysis of a single electrochemical peak, but rather to the deconvolution of the multidimensional response generated by the dual MIP–aptamer recognition interface of the analytes [242,243].

### 6.2. Chemometrics and Machine Learning

Machine learning has evolved into an operational system which resolves the difficult electrochemical sensing challenge of obtaining target molecule data from combined distorted and noisy voltametric signals. Research published during the last few years shows that four different methods exist, which include neural networks for combined identification and quantification, tree-based models which learn from unprocessed square-wave voltammetry (SWV) data, attention-based sequence models for electrochemical time series and chemometric partial least squares (PLS) methods for complex mixed substances. The methods unite to create a workable base for multi-NDSRI electrochemical detection because traditional peak-based calibration methods will not perform well.

The IdentQuantNet system operates as a drug detection solution, which developers created to detect multiple substances in cyclic voltammogram data. The workflow applies adaptive Gaussian mixture modeling to raw electrochemical data for extracting peak details, which then enter two connected systems: IdentNet for compound recognition and QuantNet for drug concentration measurement and drug interaction detection [242,244]. The framework demonstrated perfect identification precision on measured cyclophosphamide (CP) and ifosfamide (IFO) datasets while producing average absolute errors of 4.88 μM for CP and 6.67 μM for IFO [243]. The optimized model received additional training with five chemotherapeutic drugs, which included etoposide, methotrexate, ifosfamide, cyclophosphamide and 5-fluorouracil, to maintain its strong performance across both simulated and actual voltammogram data [244]. The simulation framework, which used k-means clustering, polynomial fitting and Gaussian mixture modeling, generated training data with MAPE values of 0.32% for etoposide and 4.78% for methotrexate [242]. The WeightedMSE method produced 98% non-underestimated predictions through additional optimization and the TwoWayANN model delivered 100% and 99.35% accuracy for etoposide and methotrexate within a ±5 μM error window [245,246].

Research has demonstrated that Tree-based models perform at the same level when they need to identify patterns. The Random Forest models, which used raw SWV data and discrete-wavelet-transformed signals for ten active nitro compounds, as well as their combined mixture class, achieved up to 97.3% classification accuracy throughout all concentration ranges [247]. The Random Forest and support vector machine classifiers, together with their related algorithms, have achieved between 96% and 98% accuracy for electrochemical fingerprinting of medicinal materials by analyzing both complete voltammograms and PCA-reduced features [248,249,250]. The Random Forest regression model achieved better analyte quantification performance in complex saliva matrices than linear models by producing an R^2^ 0.84 and maintaining relative errors between 19% and 25% [251].

The deconvolution process in chemometrics maintains its position as a leading method for accurate mixture analysis. The voltametric electronic tongue system, which uses three modified screen-printed electrodes, successfully measured heroin, morphine, and codeine levels together, although their signals overlapped and caffeine and paracetamol were present in the samples. The single-variable peak analysis did not succeed but PLS-1 analysis of the complete multi-electrode SWV data produced a test-set normalized RMSE of 0.084 [241]. The research applied PLS and PLS-DA methods to analyze complex electrochemical wine and plant extract fingerprints, which resulted in classification accuracies between 87% and 96% [249,252].

For the specific N-CZN/N-CZNC binary-discrimination problem, the most appropriate primary architecture is therefore IdentQuantNet, because it was explicitly designed for closely related drug signals on voltametric sensors and already combines simultaneous identification with quantitative prediction. Random Forest on DWT- or waveform-transformed SWV should serve as the secondary validation model for classification robustness, while PLS provides the chemometric baseline for interpretable linear calibration. The training approach needs to merge computer-generated N-CZN/N-CZNC voltammograms, which is created through the simulation system with actual standard measurement data that spans different concentration levels and pH values and excipient conditions [242]. The system requires machine learning as its fundamental analytical tool because future pharmaceutical electrochemical mixture analysis will depend on it to handle the various substances which interfere with the process.

### 6.3. Deconvolution for Clozapine NDSRIs

A non-selective steric signal alone cannot distinguish N-CZN from N-CZNC; successful deconvolution requires orthogonal validation against LC–MS/MS. Clozapine and its related impurity both contain secondary amine moieties susceptible to nitrosation, forming N-nitroso clozapine (N-CZN) and N-nitroso clozapine impurity C (N-CZNC) during manufacturing and storage. Because these structurally analogous tricyclic NDSRIs generate overlapping steric current-suppression profiles at an electrochemical interface, a single non-selective aptasensor or impedimetric readout cannot resolve them. Deconvolution requires anchoring the electrochemical signal to an orthogonal separation technique. Liquid chromatography–tandem mass spectrometry (LC–MS/MS) achieves baseline separation of N-CZN and N-CZNC using an Agilent Poroshell HPH C18 column under gradient elution, with a mobile phase combining 0.01 M ammonium bicarbonate in water and 0.1% formic acid in methanol:acetonitrile [223]. This chromatographic separation acts as the physical deconvolution step, isolating each NDSRI before detection.

Detection is then performed by electrospray ionization in multiple reaction monitoring (MRM) mode, tracking distinct mass transitions: *m*/*z* 356.0 → 326.0 for N-CZN and *m*/*z* 342.1 → 312.0 for N-CZNC. These distinct MRM transitions provide the definitive analyte identification necessary to calibrate any upstream electrochemical sensor. By mapping the non-selective steric current suppression to these validated LC–MS/MS outputs, researchers can build a multivariate model that distinguishes the analytes based on orthogonal mass and retention fingerprints rather than bulk interfacial crowding alone. The LC–MS/MS method exhibits excellent linearity (R^2^ > 0.999) from the limit of quantification (LOQ) to 150%, with LODs of 0.058 µg/mL for N-CZN and 0.014 µg/mL for N-CZNC. The method demonstrates satisfactory accuracy, with recoveries between 88% and 102%. Validated per ICH and USP <1225> guidelines, this approach has been successfully transferred to quality-control laboratories for routine batch analysis [223].

For AI-assisted electrochemical NDSRI quality control, regulatory acceptance will depend mainly on interpretability and cross-site robustness. Machine learning achieves its potential through the combination of standard analytical validation with explainable AI (XAI) and privacy-protecting multi-site training according to current research findings.

SHAP maintains its position as the top XAI solution, which identifies vital features and generates prediction influence maps for input data in sensing and biomedical operations [253,254]. SHAP maps for spectroscopy-like data show which spectral bands and time segments are vital for decision-making. The SHERPA CNN+SHAP framework for EEG analysis shows which specific waveform segments and channels affect decisions while linking these elements to recognized physiological components [253,255]. The research community needs to use their field knowledge to interpret SHAP and LIME explanation outputs because these methods generate misleading results when features demonstrate statistical relationships with each other [256]. The NDSRI voltametric sensing system requires a model which shows regulators that N-CZN calls stem from onset potential, shoulder shape, plateau curvature or peak asymmetry instead of an unknown latent output. The same explainability evaluation process used for photovoltaic fault detection shows that auditing has become a mandatory operational necessity [257].

Machine learning has already demonstrated that electrochemical sensors fulfill QC-style performance standards when operating in complex matrix environments. The ML-enhanced electrochemical sensing review demonstrated that artificial neural networks decreased niclosamide quantification errors because they lowered the RMSEC from 2.76 to 0.28. The DWT/FFT preprocessing method enabled the deconvolution of mixed dopamine/serotonin/ascorbate/uric-acid voltammograms which resulted in NRMSE 0.088 and R 0.974 values [258]. The ML pipeline for therapeutic-drug monitoring used Gaussian mixture modeling and neural quantification to reach 100% identification accuracy while the simulation engine produced training data with an MAPE as low as 0.32% [242,243]. The research team developed extra ANN systems which enable them to measure several drugs [245]. In biofluids, FFT-SWV plus PLS enabled simultaneous morphine, methadone, and uric acid analysis in urine with RMSECV ~0.16–0.20 μM, recoveries 96–106%, and RSDs 3.7–5.3% [259]. The studies demonstrate that ML calibration validation remains possible.

Transferability across QC laboratories is the second regulatory concern. Federated learning offers a credible solution because raw data remain local while model updates are aggregated centrally. The medical field uses federated learning between ten institutions to reach 99% of centralized-model performance by keeping patient data confidential [260]. The healthcare federated-learning system, which uses multi-party computation and additive secret sharing, achieved 97% accuracy [261] The blockchain-based federated learning system for sensor networks achieved better performance through enhanced accuracy and operational efficiency and network security protection [262]. The MELLODDY consortium demonstrated that cross-company federated learning of more than 2.6 billion confidential assay data points improved QSAR prediction and applicability domain without exposing proprietary datasets [263].

To satisfy a pharmaceutical regulatory pathway, this trust framework should be explicit. Under ICH Q2(R2), the AI-enabled electrochemical procedure would still need to demonstrate specificity, linearity, accuracy, precision, detection capability, quantitation capability, range, and robustness, while ICH Q14 provides the broader science- and risk-based framework for analytical procedure development and lifecycle management. The SHAP-based importance maps demonstrate that the model uses N-CZN and N-CZNC voltametric features for its responses instead of generic matrix-related signals. The PAT framework from FDA supports this approach because it promotes the use of scientific sensor-based systems for quality control during pharmaceutical manufacturing and development through at-line and in-line monitoring (USFDA PAT 2018). Machine learning creates no reduction in regulatory confidence because it allows for better electrochemical analysis of N-CZN and N-CZNC, which makes it both more transparent and easier to apply in practice.

## 7. Hybrid Integrated Sensor Architecture

### 7.1. Rationale for MIP–Aptamer Dual Recognition

For bulky tricyclic NDSRIs such as N-nitroso clozapine (N-CZN), neither molecularly imprinted polymers (MIPs) nor aptamers alone fully satisfy the combined requirements of affinity, selectivity, stability, and matrix tolerance. The dual-recognition studies which emerged recently demonstrated that MIP–aptamer dual-recognition systems solve the individual problems which each format possesses to create an enhanced detection method for complex rigid tricyclic molecules of approximately 300–450 Da.

The main limitation of MIP-only systems is their structural heterogeneity. The bulk imprinting process creates particles which display irregular shapes and varying sizes, while their cavities become partially hidden, their swelling patterns are uneven and their mass diffusion rates are slow, which leads to decreased availability of active sites and higher levels of non-target material adsorption [264,265]. The research community identifies two essential obstacles which block ultra-trace sensing and commercialization because they trigger non-specific binding, produce shallow or ill-defined cavities and generate poor uniformity when analyzing complex matrices [29,266]. The problem deepens when analyzing big molecules which perform multiple functions because basic monomer-based imprinting lacks sufficient binding power to identify similar molecules [266]. Real-world samples demonstrate this effect that MIP sensor functionality for neuron-specific enolase detection deteriorated when used with serum instead of buffer solution due to protein interference, which blocked the imprinted sites. The distribution patterns of tricyclic NDSRIs suggest that cavities exist at depths which prevent scientists from distinguishing between N-CZN and structurally related nitrosamine compounds and their precursor impurities.

The aptamer-only systems deliver improved molecular discrimination but their performance deteriorates at the solid interface. The use of aptamers is limited because they degrade through heat, chemical reactions and enzyme breakdown [267]. The Apta-MIP reviews show that aptamers which remain exposed will undergo denaturation, fouling and functional loss when used with actual samples, which leads to decreased measurement precision and reduced detector responsiveness [267,268,269]. The immobilization process requires various approaches because research demonstrated that different coating methods, including dip-coating, drop-casting and covalent attachment, result in distinct detection limits and linear response ranges for identical recognition elements [270]. The research also demonstrated that loading too many aptamers onto hybrid interfaces results in decreased electrical response because the extra surface material creates physical barriers which block movement and cause steric hindrance [267].

MIP–aptamer dual-recognition sensors successfully eliminate the opposing weaknesses their individual components produce. The impedimetric tetracycline sensor employs an aptamer whose MIP layer encapsulates to create two recognition sites, which achieved a detection limit of 144 fM throughout the detection range of 0.5–100 pM and 1–1000 nM while achieving better results than sensors which used only one receptor type [271]. The magnetic MIP–aptamer–β-cyclodextrin platform for tetracycline separation showed an imprinting factor of 7.6 and recoveries of 85–111.5% in animal feed, which proved their better selectivity and matrix tolerance [18]. The dual-mode dibutyl phthalate platform which uses MIP–aptamer recognition with nanoenzyme amplification achieved LODs of 0.263 nM in photoelectrochemical mode and 30.1 nM through colorimetric detection because of its combined recognition system [272]. MIPs provide mechanical and chemical stability, as shown in multiple reviews, which also state that aptamers offer precise target recognition and a binding capability that MIPs lack to produce a true “1 + 1 2” effect [18,267,268,269,273].

MIP–aptamer dual-recognition sensors function as a fundamental solution for bulky NDSRIs such as N-CZN. The MIP serves as the initial identification and sample concentration unit, which filters substances before the aptamer performs the second stage of detection to differentiate between N-CZNC and similar contaminants.

### 7.2. Quantified Dual-Recognition Synergy

Recent dual-recognition studies show that MIP–aptamer dual-recognition sensors consistently outperform single-layer formats in both sensitivity and selectivity, supporting their use for highly similar NDSRIs such as N-CZN and N-CZNC. The MIP provides preconcentration and size/shape filtering and interfacial strength while the aptamer enables detailed molecular detection and improved signal generation [18,268,269,273,274].

The strongest proof emerges from systems that researchers evaluated against corresponding aptamer-only and MIP-only versions. The Apta-MIP platform in bacterial impedimetric sensors achieved detection limits of 4 CFU/mL for S. aureus and 2 CFU/mL for E. coli within a 1–10 CFU/mL detection range; the authors credited its success to the dual-recognition system, which combined aptamer binding with imprinted cavity recognition [20]. The Apta-MIP system achieved a prostate-specific antigen detection limit of 1 pg/mL, which represented a threefold improvement in sensitivity compared to the aptamer-only receptor, and simultaneously reduced false-positive responses from proteins including hK2 and HSA [275]. The trypsin system achieved better binding through hybrid aptaMIP nanoparticles, which lowered the detection limit from 4.1 nM to 2.4 nM and enhanced affinity from 12.3 × 10^−9^ M and 10.3 × 10^−9^ M to 6.8 × 10^−9^ M [276]. These are not marginal differences; they demonstrate quantitative synergy.

Small molecules experience identical sensing patterns. The dual-recognition MIP–aptamer electrochemical sensor that uses CS-MWNT/AuNPs for chloramphenicol detection achieved an LOD of 3.3 × 10^−12^ g/mL throughout the 10^−12^–10^−6^ g/mL concentration range [12]. The impedimetric chloramphenicol apta-MIP system delivered an improved LOD of 0.3 pM throughout the 1 pM–1 nM detection range because of its excellent selectivity, which results from MIP and aptamer recognition systems that use the “double exact property” [277]. Histamine was similarly detected at 0.11–0.15 nM using a dual-recognition aptamer/MIP sensor on AuNP-decorated carboxylated CNTs, with strong selectivity in plasma and canned tuna matrices [278]. The hybrid design achieved a detection limit of 0.31 fg/mL for leptin, improving on the previous range of 1 fg/mL–10 pg/mL. The method achieved sample recovery rates between 106% and 109% for both serum and plasma samples [279]. In another example, a cyt c aptamer–MIP/quantum-dot platform produced an imprinting factor of 3.17 and selectivity factor of 8.33 [280].

More complex targets confirm this principle. Ni_3_(BTC)_2_ MOF-modified SPCEs served as the foundation for a SARS-CoV-2 MIP–aptamer sensor, which demonstrated an LOD of 3.3 PFU/mL between 10 and 10^6^ PFU/mL concentrations while providing superior sensitivity and improved real-world testing results compared to MIP-only and aptamer-only control systems [281]. For aflatoxin B1 detection, researchers have developed several aptamer–MIP systems which achieve pg/mL-level sensitivity while maintaining the ability to operate without interference and perform self-calibration through the combination of porous COF/MOF-based MIP substrates with aptamer recognition [274,282,283,284]. Research on dopamine and gliadin dual-recognition systems demonstrates that MIP functions as a molecular filter, which enhances sample quality before the aptamer performs its specific function to trigger signal changes [285,286]. The dual-mode platform which uses MIP–aptamer recognition with nanoenzymes achieved detection limits of 0.263 nM through photoelectrochemical analysis and 30.1 nM through colorimetric analysis [272].

These research findings establish a practical baseline for N-CZN/N-CZNC detection because dual-recognition systems demonstrate the ability to achieve detection limits which are three to ten times better than single-receptor systems while maintaining selectivity values above eight to ten. The current review uses this projection to demonstrate why researchers should select hybrid receptors instead of choosing between aptamer-based and MIP-based receptor systems.

### 7.3. Integrated MIP–Aptamer Sensor Blueprint

A practical electrochemical sensor for bulky N-nitroso drug substance-related impurities such as N-nitroso clozapine (N-CZN) must simultaneously address three problems: the heterogeneous cavities in conventional MIPs, the instability of surface-bound aptamers, and matrix interference in real samples. The hybrid system combines dual-monomer electropolymerized MIPs with embedded aptamers and nanostructured conductive transducers, which were mechanically validated in previous studies [269,273]. Figure 4 shows the layered architecture of the MIP–aptamer dual-recognition sensor, which operates through a signal-off mechanism and achieves the predicted analytical results that will serve as a functional prototype for developing the N-CZN sensing platform.

The proposed sensor consists of a 3D-printed scaffold with pores, an activated conductive filament, a Ti_3_C_2_T_x_ MXene/AuNP/cCNT transduction layer and a dual-monomer electropolymerized MIP, which includes a thiolated aptamer within its structure. The porous scaffold enhances analyte access while the MXene–nanocarbon layer enables faster electron transfer, and the MIP–aptamer dual-recognition interface with micropores smaller than 2 nm uses two recognition methods to detect targets through their shape and their specific interaction with aptamers. The redox-probe becomes inaccessible because of N-CZN or N-CZNC binding, which forms a physical barrier that blocks electron transfer, thus causing a signal-off response. The projected performance dashboard summarizes the anticipated sensitivity, selectivity, matrix recovery, regeneration, and greenness outcomes.

The recognition stage requires a basic conducting copolymer which combines pyrrole with thiophene-type monomers to achieve optimal performance because dual-monomer MIPs achieve better results than single-monomer systems. The research shows that selectivity factors rise from 2.0 to 2.4 in mono-monomer MIPs to 3.7–4.4 in dual-monomer designs through complementary interaction design. The rigid tricyclic scaffold of N-CZN needs both π-stacking and directional interactions for its tricyclic scaffold, which makes this subject important. The magnetic MIPs, which target tricyclic analogs including cyproheptadine, amitriptyline and ketotifen, showed imprinting factors of 4.74–6.91 and adsorption capacities of 42–49 mg g^−1^ and 94.8% recoveries during water testing, which proved that precisely designed cavities can identify large aromatic molecules effectively. The film receives thiolated aptamer integration, which creates molecular selectivity and defends the nucleic-acid receptor against the contamination and breakdown that occur in aptamer-only systems [267].

The transduction layer should be equally deliberate. AuNP-decorated carboxylated CNTs serve as a conductive scaffold with high surface area which allows aptamer attachment and precise molecularly imprinted polymer (MIP) development. The detection limits of dual-recognition systems which use AuNP/cCNT-based surfaces reach extremely low levels: 0.11–0.15 nM for histamine in plasma and food matrices [278] and 0.3 pM for chloramphenicol with strong interferent resistance [277]. The nanostructured substrate functions to enhance interfacial changes instead.

Dual-recognition systems deliver superior results when compared to systems which use only one control layer. The aptamer–MIP receptor in PSA sensing achieved an LOD of 1 pg mL^−1^, which proved to be three times more sensitive than the aptamer-based version [275]. The trypsin detection system using aptaMIP nanoparticles achieved better affinity than nanoMIP and aptamer alone by reaching 6.8 × 10^−9^ M and also improved the detection limit from 4.1 nM to 2.4 nM [276]. Another study developed a hybrid aptamer–MIP/QD protein sensor, which achieved an imprinting factor of 3.17 and a selectivity factor of 8.33 [280]. The magnetic aptamer–MIP system which tolerates matrices achieved recovery rates between 85% and 111.5% for complex feed samples. N-CZN validation using spiked plasma or wastewater should produce recovery results between 85% and 115% with an RSD <8% [120].

The construction of thin electropolymerized films makes template removal easier because these films provide better access to the surface. Acidic methanol-based extraction together with electrochemical regeneration methods help maintain the original shapes of electropolymerized MIP cavities. Green thiophene-based electro-MIPs demonstrate that microliter-scale monomer volumes and aqueous-rich media can be used without sacrificing analytical performance [95]. A complete manufacturing process needs to start with substrate conductivity preparation, before applying surface modifications which support aptamer binding, and then performing dual-monomer MIP formation through controlled cyclic–voltametric operations with N-CZN templates, and finally ending with template removal through MeOH/AcOH-assisted treatment and final electrochemical conditioning. The research data supports N-CZN as a valid platform which should achieve sub-picomolar to low-picomolar sensitivity and selectivity factors above 8, work with actual samples, and maintain its functionality after multiple regeneration processes, although the N-CZN-specific aptamer and final receptor geometry require experimental confirmation. The projected analytical performance and risk assessment relative to benchmark analytes are summarized in Table 5.

### 7.4. Discrimination of N-CZN from Clozapine and N-CZNC at Pharmaceutical Excess Ratios

The proposed MIP/aptamer architecture provides a plausible route to discriminate N-CZN from both parent clozapine and N-CZNC because dual recognition can combine cavity-level steric and interaction complementarity with the higher molecular specificity of an aptamer [18,269,273]. This mechanism is consistent with electrochemical AptaMIP studies showing that target rebinding decreases redox-probe current through restricted electron transfer, while the hybrid format improves sensitivity, affinity, and stability relative to either recognition element alone [12,268,278]. However, selectivity at pharmaceutical excess ratios should not be inferred from single-analyte experiments or from structurally related interferents tested separately, because MIP selectivity is difficult to interpret without mixed-solution competition data [290]. The critical validation experiment is therefore competitive rebinding in the presence of large clozapine excess and added N-CZNC, benchmarked against NIP controls and orthogonal LC–MS/MS quantification. Until those data are shown, the sensor appears mechanistically credible for N-CZN discrimination, but the evidence remains provisional for true impurity-resolved quantification under formulation-relevant conditions [291,292,293].

### 7.5. Limitations and Failure Modes

Despite the strong conceptual promise of the proposed 3D–MXene–MIP–aptamer architecture, several practical failure modes must be considered before translation to routine pharmaceutical analysis. First, although porous electrodes can improve analyte access and internal surface area, they remain vulnerable to surface fouling and pore blocking, which can increase background noise, suppress signal magnitude, and reduce sensitivity, reproducibility, and long-term reliability [294,295,296,297]. This concern is especially relevant for bulky tricyclic analytes and complex pharmaceutical matrices, where partial pore obstruction or nonspecific adsorption could convert the intended steric signal-off mechanism into poorly reversible electrode passivation.

A second limitation is that fouling may not be fully reversible. The long-term sensor literature distinguishes reversible fouling, which can be removed by routine conditioning, from irreversible fouling, which requires stronger mechanical, chemical, electrochemical, or integrated cleaning procedures and may still compromise baseline recovery and sensor lifespan [298]. Accordingly, regeneration claims for the proposed platform should be framed not only in terms of signal recovery after one cycle, but also in terms of repeated-cycle durability, baseline drift, and retention of selectivity after cleaning.

A third failure mode concerns the stability–sensitivity tradeoff of Ti_3_C_2_T_x_ MXene. The attached review emphasizes MXene as a high-surface-area, high-capacitance transduction layer, but prior electrochemical work shows that exposure of Ti_3_C_2_T_x_ to anodic potentials in aqueous media oxidizes the material toward TiO_2_, making it less electrochemically active over time [299]. For this reason, short-term signal amplification should not be interpreted as evidence of long-term analytical stability unless oxidative drift is measured against the magnitude of the analytical response. More recent MXene studies show that this limitation can be mitigated, but not ignored, through stabilization strategies that inhibit leaching, restacking, or oxidation, including ionic-liquid confinement and noncovalent organic interactions [300,301,302].

A fourth limitation concerns recognition selectivity. The proposed hybrid interface is attractive precisely because MIPs alone can suffer from limited specificity, while aptamers alone can lose stability in harsh environments [269,300,303]. MIP–aptamer dual-recognition systems generally improve selectivity, affinity, and stability relative to single-recognition formats, but that advantage should be demonstrated experimentally rather than assumed [18,269,273,274]. This is particularly important because selectivity tests for imprinted systems can be misleading when comparator conditions are not rigorous, and because the manuscript’s intended application involves discrimination among closely related bulky species rather than unrelated interferents alone [295].

Taken together, these limitations suggest that future validation of the proposed platform should explicitly quantify fouling resistance, reversibility of regeneration, MXene drift under operating potentials, and the selectivity gain of dual recognition over MIP-only and aptamer-only controls. Such a framework would strengthen the translational credibility of the proposed sensor and clarify the operational boundaries within which porous 3D–MXene architectures are likely to outperform planar or single-receptor alternatives [294,295,298,304].

## 8. Discussion

The research findings demonstrate that tricyclic NDSRI detection problems stem from translation issues instead of material-based challenges because the most challenging analytes lack sufficient validated detection methods and depend only on LC-MS/MS analysis systems. Their assessment requires nitrosamine detection systems to determine their limitations in handling complex tricyclic NDSRIs, to ensure reproducibility, data quality, regulatory approval and successful field application [305,306].

The most significant gap is that tricyclic NDSRIs lack target-specific validation data, whereas smaller nitrosamines already have multiple quantitative methods across water, food, and tobacco matrices [307,308,309]. This research gap arises due to the lack of established reference standards for NDSRIs, limited isomer diversity, structural similarities with parent APIs, matrix ion suppression, and artifacts’ formation, which present challenges during sample processing [306]. Current evidence indicates that the success of NDMA or TSNA cannot be used as proof for clozapine-like impurities since no electrochemical MIP, aptamer, ECL or CRISPR platform exists for tricyclic NDSRI detection.

### 8.1. Comparative Challenges

The LC-MS/MS-based analytical approach has always remained the gold standard in providing reliable quantitative results through selective enrichment integration, though most findings derive from basic nitrosamine research rather than pharmaceutical NDSRI analysis [306,310]. The MIP-SPE HPLC-MS/MS method achieved recovery rates of 93–107% in water and beverage samples, detecting substances at 0.2 to 0.7 ng/L with a measurement precision between 4.9 and 10.5% [307]. The dummy-template MIP extraction method produced processed-meat results which showed R^2^ values above 0.9985m, detection limits between 0.03 and 0.65 ng/g and measurement precision ranging from 1.20 to 9.76% [138]. The LC method, which used MIP-packed columns and followed FDA-oriented guidance, achieved detection limits of 0.03 ng/mL and quantification limits of 0.1 ng/mL, produced results with 91.8–110% accuracy and less than 15% precision error, and maintained sample stability through 72 h of autosampler operation [309]. Biosensors employing electrochemical methods and signal amplification techniques exhibit superior detection capabilities compared to other biosensors. These biosensors still have not reached their full potential in analytical development and they still require further testing in diverse pharmaceutical sample matrices [311]. The NDMA MIP sensor system delivers detection limits of 1.16 ppb through 18 regeneration cycles. In contrast, the CRISPR-ECL NDMA platform detects 5.33 pg/mL concentrations of NDMA within the concentration range of 10 pg/mL to 10 μg/mL [21]. The DNA-carbon-dot sensor demonstrated detection capabilities for NDMA and NDEA within concentration ranges from 9.6 to 9.9 × 10^−9^ M. The aromatic dephostatin biosensor can detect NDMA within a limit of 0.029 nM with 2.5% reproducibility. Furthermore, the biosensor produced results identical to HPLC when testing food samples [25,312]. The evidence demonstrates that none of these technological improvements have resolved the problem of recognizing bulky tricyclic nitrosamines because their large size, poor electrochemical properties and insufficient detection methods continue to be major challenges.

### 8.2. Validation, Regulation, and Implementation

This research study revealed clinical and matrix validation as the least effective study domain. This research demonstrated that no tricyclic NDSRI detection system has obtained validation yet. The entire aptasensor field faces two main obstacles: insufficient population-based clinical trials and sensors which fail to operate properly in biological environments [313]. Translation papers demonstrate that biosensors need to show reproducible detection of target molecules in relevant samples for field deployment instead of achieving high buffer sensitivity, but most biosensor research uses diluted samples for short-term testing and single-use demonstrations, which produce insufficient data for field deployment [311]. The dataset itself creates problems which affect the ability to reproduce the results. The field lacks standardized reference materials for many NDSRIs, and the compounds themselves show isomer complexity, parent-drug overlap, and a high API background that obscure weak impurity signals [306]. These existing restrictions become more severe because biosensors have different levels of performance due to sample matrix effects, environmental factors, user interactions and the absence of uniform manufacturing standards followed by multiple laboratories [314,315]. The current rate of regulatory change surpasses the development of analytical standards. The ICH M7 and FDA NDSRI guidance provides a risk framework but does not address the ultra-trace-level detection of bulky tricyclic impurities. Acceptable daily intake of NDSRIs depends on read-across and CPCA evaluation, which remains a temporary solution pending further data [316,317]. Research shows that the NMPEA limit evaluation became more severe when increasing the AI from 8 ng/day to 40.1 ng/day, which demonstrates how insufficient potency data affects the establishment of control limits for nitrosonortriptyline [318]. Health authorities have also challenged the reliance on ICH M7 Option 4 purge rationales for nitrosamines without confirmatory analytical testing, noting the underestimation of impurity levels [319]. The actual application of this chemical process faces numerous obstacles which reach beyond the field of chemistry. The translation of biosensors requires standardization, together with scalable fabrication, training and infrastructure, data management, interoperability, privacy protection and cybersecurity measures, because AI-assisted analysis and connected devices need these elements [311,314,315]. The research needs to address four essential knowledge gaps, which include laboratory-based external reproducibility testing, the development of validated NDSRI reference standards, method comparison studies between new approaches and official LC-MS/MS techniques for finished drug products, and the assessment of long-term product stability under actual matrix conditions [311]. The current detection methods enable ultra-trace nitrosamine identification but the research shows that tricyclic NDSRIs have not yet received validation; scientists need to move past theoretical sensor development through proper comparative testing, standardized databases and proof of operational readiness.

## 9. Conclusions

Tricyclic NDSRIs present an advanced analytical challenge because these impurities combine four major obstacles, which include their large size, strong sample interference, and weak electrochemical response, as well as the absence of proven detection methods. The central insight of this review is that the effective, field-deployable monitoring of N-nitroso-clozapine-like contaminants cannot be achieved by incremental improvements to LC–MS/MS; instead, it demands purpose-built transduction architectures that integrate porous transport networks, molecular-level selectivity, nanomaterial-enhanced signal generation, and computational signal deconvolution.

Sensor Rationales, Supported by Analog Data: Electrochemical sensing has established itself as a dependable main monitoring solution, which now competes with LC–MS/MS instead of functioning as a supplementary method. The three-dimensional porous electrodes enable rigid tricyclic analytes to overcome their steric-blocking effect because the 3D-printed MXene microlattice structure produces a 10 nM detection limit with response times of under 10 s and a 1000-fold improvement in sensitivity compared to planar Ti_3_C_2_T_x_ films. The pore-engineered architectures enable better analyte access, which leads to faster reaction times. The design of conductive filaments determines the level of electron-transfer performance and the material’s ability to move between locations. The standard heterogeneous rate constant k^0^ for activated CNT/CB/PLA electrodes reaches 5.02 × 10^−4^ cm s^−1^. The production of these electrodes takes 3.7 min, while their material expenses total $0.11 for every electrode produced. The manufacturing of these electrodes uses recycled graphite/CB/PLA filaments which do not need any activation after printing (k^0^ = 1.26 × 10^−3^ cm s^−1^, LOD 5.7 µM, cost £0.05). The MXene (Ti_3_C_2_T_x_) transducers operate as multilayer conductors, which have an extensive surface area while their –O/–OH/–F terminal groups enable aptamer immobilization and achieve sensitivity levels in the femtomolar range. The MIP–aptamer hybrid recognition elements solve the selectivity problem, which MIPs and aptamers fail to solve when used separately. The imprint-defined cavity creates shape-matching through its design, yet the aptamer unit enables target binding and produces detection signals, resulting in dual-recognition systems.

Best-Performing Analog Platforms: The performance standards of tricyclic NDSRIs remain unknown because no electrochemical sensor has received validation but similar systems provide actual performance measurements. An electrochemical sensor which combines chloramphenicol MIP–aptamer technology detects concentrations down to 3.3 × 10^−9^ g L^−1^ within a sensor detection range between 10^−8^ and 10^−2^ g L^−1^. The dual-recognition sensor for aflatoxin B1 achieved a detection limit of 2.31 × 10^−4^ ng mL^−1^ while a previous AFB1 hybrid showed a detection limit of 12.0 pg L^−1^. The detection of dibutyl phthalate through hybrid multi-signal platforms achieved a limit of detection at 0.263 nM by using photoelectrochemical methods, while the orthogonal dual-signal approach reached a limit of 0.648 pM. The Ti_3_C_2_T_x_ aptasensor for chloramphenicol detection using MXene technology demonstrated a detection range from 10 fM to 1 µM and achieved a detection limit of 1 fM while successfully detecting milk samples. The obtained figures of merit, which involve small molecular targets with matching dimensions and water-repelling properties, are expanded on in the following.

SWOT Summary of Translation: The current state of tricyclic NDSRI sensor translation becomes visible through a SWOT analysis. Strengths include portable, low-cost electrochemical formats, MXene conductivity and facile functionalization, MIP–aptamer dual selectivity, and 3D-porosity-enhanced kinetics. Weaknesses exist because tricyclic NDSRI aptamers lack experimental proof and scientists still face difficulties with matrix selection and the consistency of results, there is insufficient data from actual samples, and the production process creates different MXene structures through restacking. Opportunities lie in microfluidic integration, wearable/portable devices, multiplexed detection, AI-assisted signal deconvolution, and greener quality-control workflows. Threats center on the fact that regulatory acceptance remains firmly tied to validated LC–MS/MS; the structural similarity between NDSRIs and their parent APIs still complicates orthogonal confirmation, and creates the risk of false-positive results.

Future Directions and Closing Perspective: The transition from theoretical sensor development to working experimental systems has three specific priorities which must be addressed right away. The development of target-specific recognition chemistry requires two methods, computational aptamer selection and NDSRI-based molecular imprinting, because all current MIP–aptamer achievements come from food and environmental sample testing. The most reliable transducer direction appears to be stabilized MXene–3D integration, which uses 3D printing to create structures that permit physical access and MXenes to provide electrical conductivity and diverse surface-attachment options. The hybrid electrode concept was proved through the use of MXene quantum-dot-modified 3D-printed graphene/PLA electrodes, which achieved a dopamine detection limit of 3 nanomolars. Future research needs to implement regulatory-style validation methods which should use authentic pharmaceutical substances, including tablets and capsules, plasma and wastewater, and products under stress. The current NDSRI analytical standards rely on validated parameters, which include accuracy, linearity, robustness and matrix-specific calibration, and LC-MS/MS methods can detect substances at concentrations between 0.05 and 0.8 nanograms per milliliter and 0.006 parts per million.

The current systems provide solutions for separate components of the tricyclic NDSRI puzzle but they do not address the entire problem. The LC–MS/MS platform stands as the sole validated method for detecting pharmaceutical NDSRIs at levels which regulators consider trace. The combination of porous 3D electrodes, MXenes and MIP–aptamer hybrids creates a solution which overcomes transport and steric restrictions while providing better selectivity than individual-recognition sensors. The most defensible conclusion is therefore that a converged platform—combining a 3D-printed porous architecture, MXene-based amplification, computationally evolved MIP–aptamer dual recognition, and AI-assisted signal deconvolution—appears to be the most promising route for routine, on-site monitoring of N-nitroso-clozapine and related tricyclic NDSRIs. The field needs to prove this integrated sensor system through testing with actual target samples because this validation step represents the primary advancement which scientists need to make in their research.

## Figures and Tables

**Figure 1:**
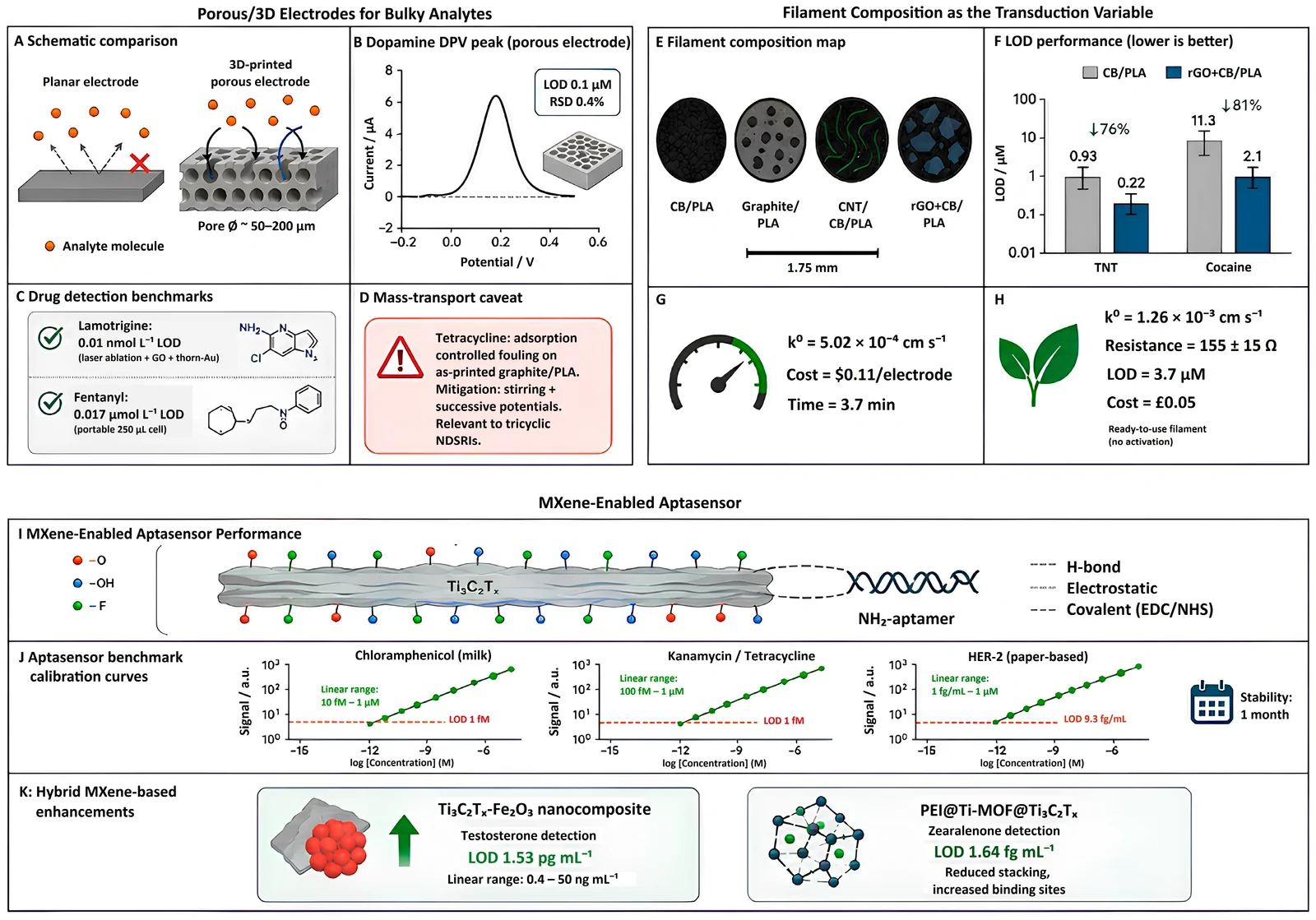
Representative porous 3D-printed, conductive-filament, and MXene-enabled transduction platforms for bulky tricyclic NDSRIs. (**A**–**D**) Planar versus porous electrodes, highlighting increased surface area, improved analyte transport, representative bulky-analyte benchmarks, and mass-transport limitations. (**E**–**H**) Effects of filament composition on sensitivity, electron-transfer kinetics, activation, cost, and sustainability. (**I**–**K**) Ti_3_C_2_T_x_ MXene surface chemistry, aptamer functionalization, representative performance, and hybrid strategies to reduce restacking and increase binding sites.

**Figure 2:**
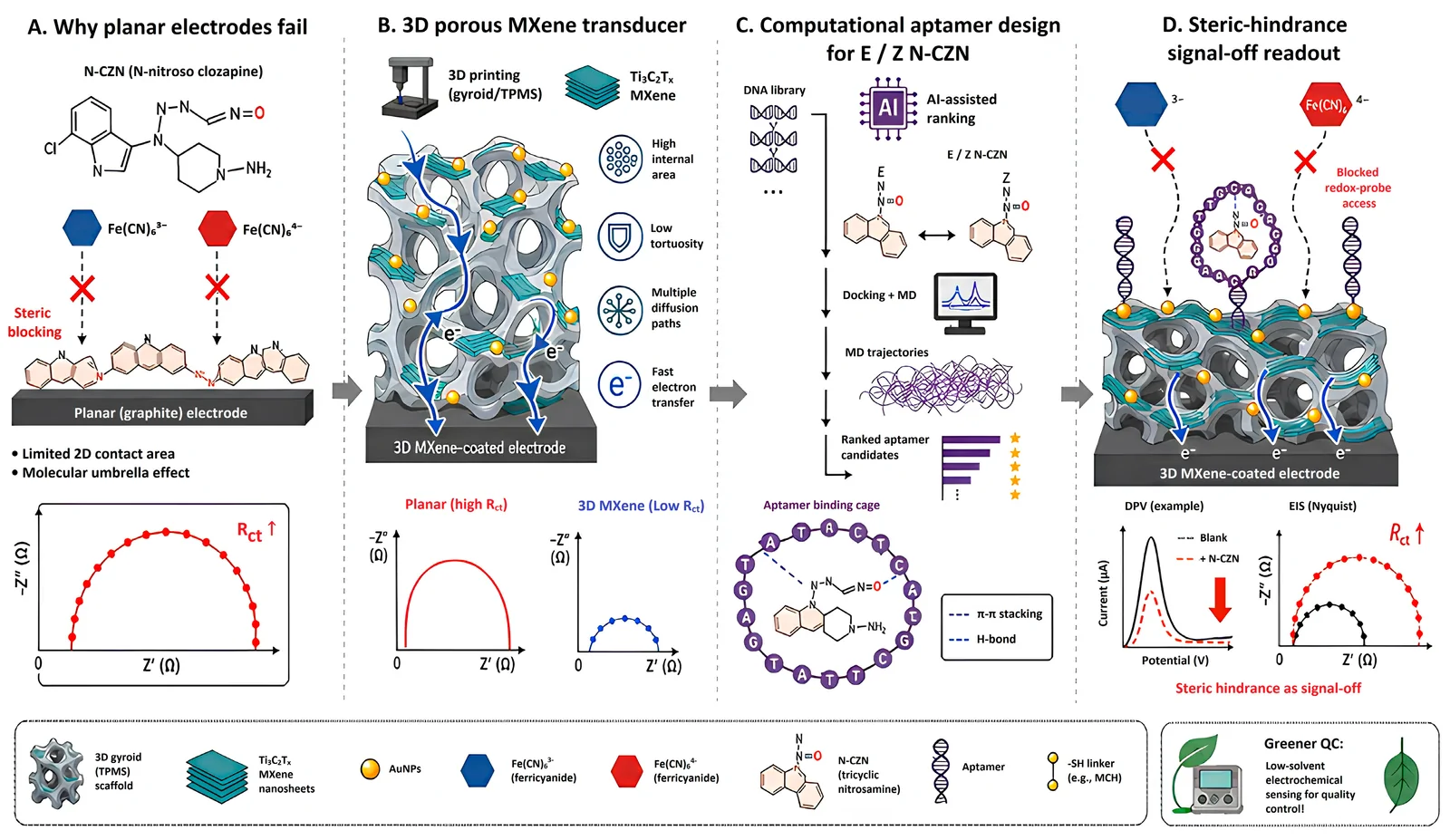
**Schematic comparison of planar versus 3D porous transducer architectures for tricyclic NDSRI detection.** (**A**) Planar graphite electrodes are susceptible to the “molecular umbrella effect,” where sterically constrained tricyclic analytes like N-CZN cause steric blocking and increase charge-transfer resistance. (**B**) The proposed 3D porous gyroid scaffold, modified with Ti_3_C_2_T_x_ MXene and gold nanoparticles, overcomes this limitation by providing a high internal surface area and accelerated electron transfer. (**C**) The recognition element relies on in silico aptamer design, utilizing molecular dynamics and AI ranking to account for E/Z isomerism in targets like N-CZN. (**D**) The final integrated platform utilizes a steric-hindrance signal-off mechanism, where target binding blocks redox probes from accessing the porous network, enabling sensitive detection.

**Figure 3:**
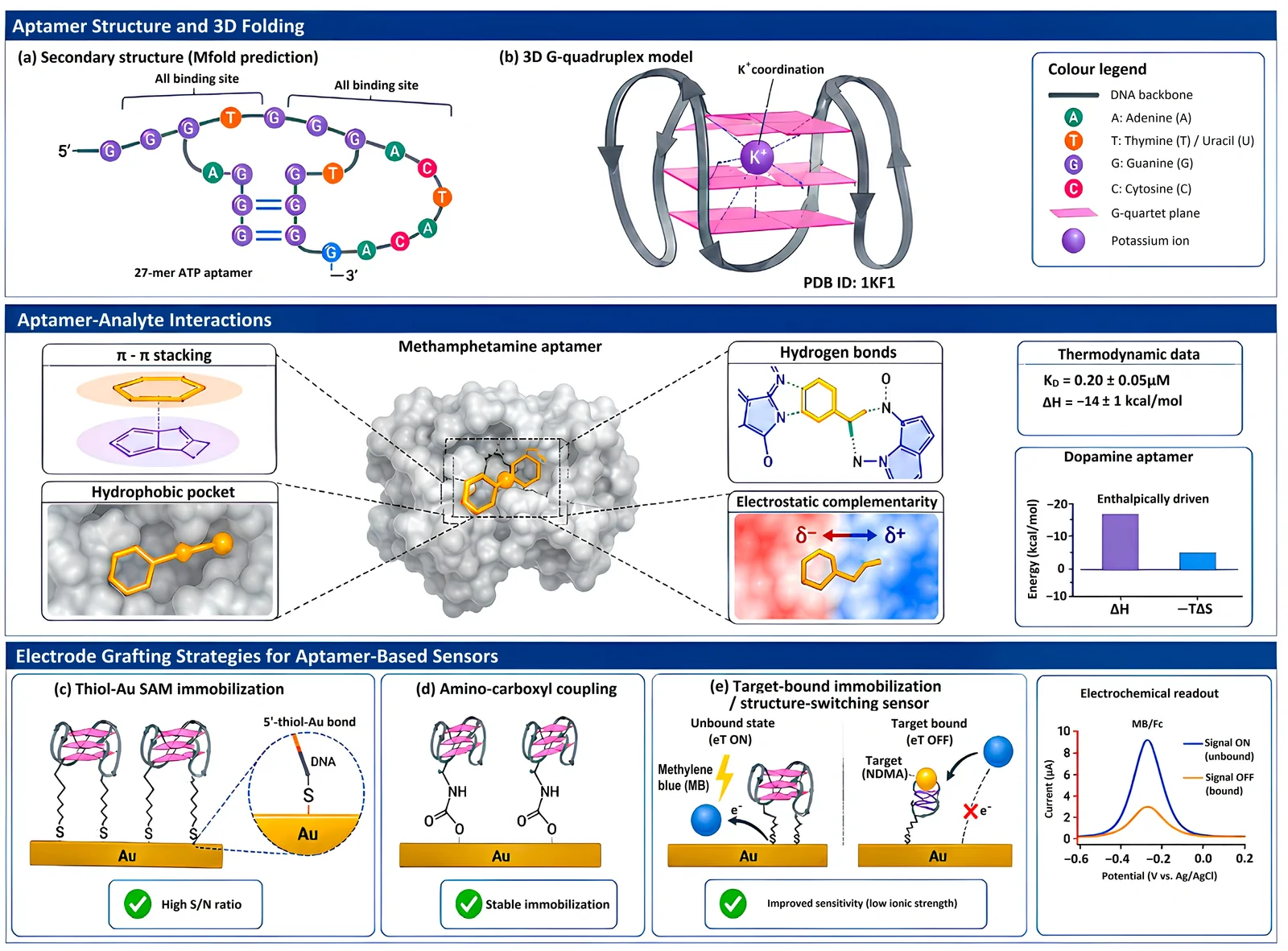
Structural, molecular-recognition, and electrode-integration principles supporting aptamer engineering for tricyclic NDSRIs. (**a**,**b**) Representative secondary and three-dimensional aptamer structures forming small-molecule-binding pockets. Key recognition interactions (middle panel) include π–π stacking, hydrophobic interactions, hydrogen bonding, and electrostatic complementarity; the ATP, methamphetamine, and dopamine examples are mechanistic precedents, not validated N-CZN aptamers. (**c**,**d**) Common immobilization strategies, including (**c**) thiol–gold bonding and (**d**) amino–carboxyl coupling. (**e**) Conceptual signal-off transduction (structure-switching format), where target binding reduces electron transfer and current.

**Figure 4:**
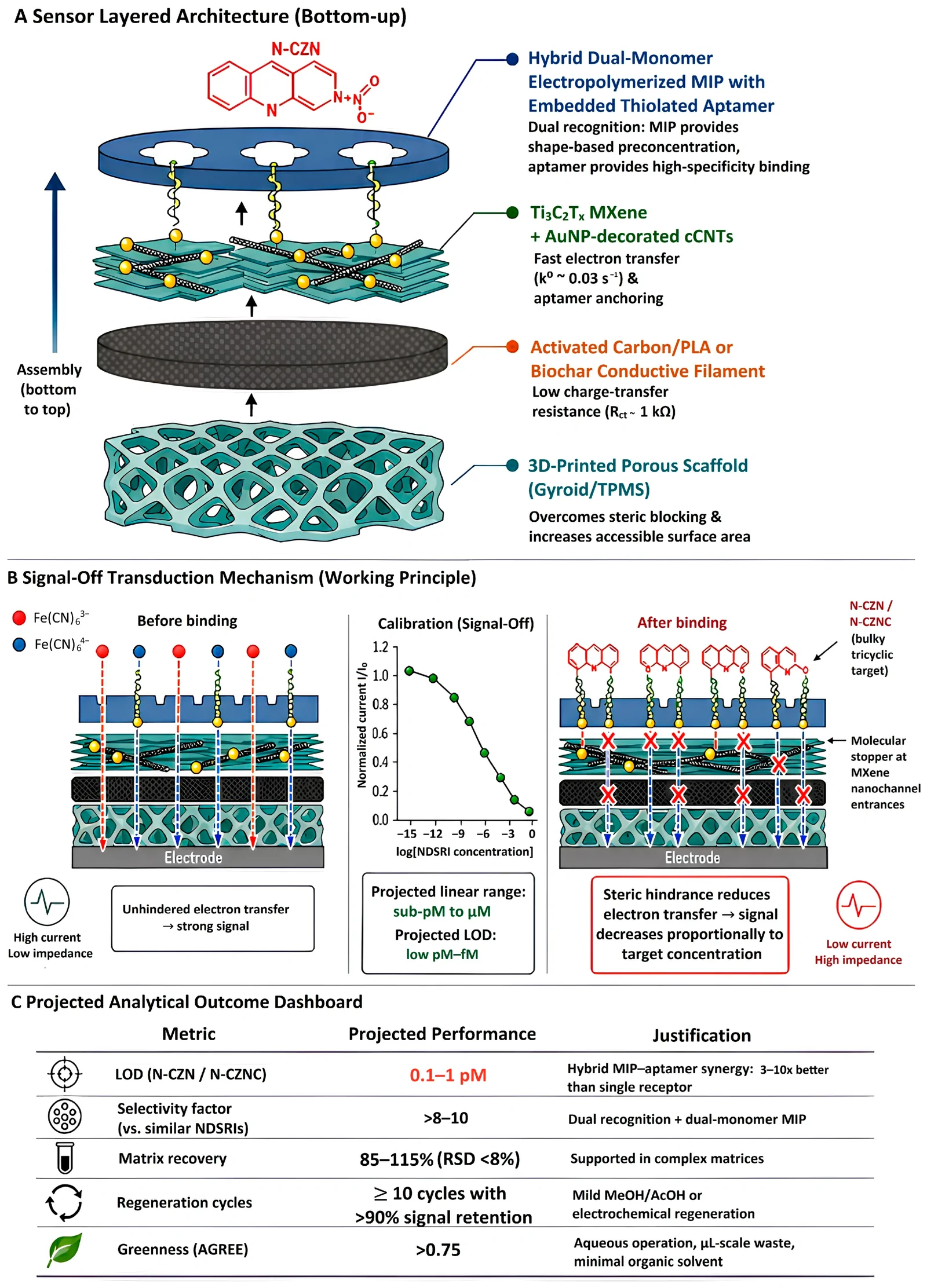
**Layered architecture and signal-off transduction mechanism of the proposed hybrid MIP**–**aptamer sensor.** (**A**) The bottom-up assembly integrates a 3D-printed porous scaffold, a conductive carbon/PLA filament, and a Ti_3_C_2_T_x_ MXene/cCNT layer to maximize electron transfer. The top layer features a dual-monomer electropolymerized molecularly imprinted polymer (MIP) embedded with specific thiolated aptamers to achieve synergistic target recognition. (**B**) In the absence of the analyte, redox probes freely diffuse through the open nanochannels, yielding a strong current. Upon binding of bulky tricyclic targets (N-CZN/N-CZNC), the nanochannels are physically blocked, resulting in a proportional signal decrease and increased impedance. (**C**) Projected analytical metrics highlight the platform’s potential for sub-picomolar detection, high selectivity, and alignment with green analytical chemistry standards.

**Table 1:** 3D-printed and MXene-enabled transduction platforms: quantitative performance and implications for bulky tricyclic NDSRIs.

Transduction/Architecture Strategy	Representative Platform	Reported Performance Metrics	Interpretative Significance and Limitation	References
Voltammetric/activated 3D-printed carbon	Post-processed FDM CNT/CB/PLA electrodes	LOD: 1.45 µM (dopamine); k^0^ = 5.02 × 10^−4^ cm s^−1^	As-printed electrodes remain surface-limited and insulated by polymer binders, requiring electrochemical or chemical activation to expose conductive materials	[142,143,144,145]
Voltammetric/TPMS and gyroid porous architectures	3D-printed lattices and gyroid scaffolds	Porosity ~80% maintained with structural integrity	High open porosity prevents steric blocking of sterically constrained tricyclic analytes, making electrode geometry as critical as conductivity	[146,147]
Impedimetric/porous electrode impedance reduction	DLP-printed porous SnO_2_ electrode	Charge-transfer impedance ~1/25 of planar electrodes	Lower interfacial resistance offsets slow electron-transfer caused by bulky aromatic analytes, though nitrosamine validation is absent	[134]
Amperometric/flow/topology-optimized 3D electrodes	3D-printed carbon lattice flow electrodes	16% decrease in power requirements vs. homogeneous porosity	Improved mass transfer sustains higher current under transport-limited conditions for low-concentration sensing	[83]
Voltammetric/3D-printed bulky molecule sensing	Printed electrodes for atorvastatin, steroids, purines	Successful detection of organics larger than simple ions	Indirect feasibility evidence for bulky pharmaceutical impurities, though rigid tricyclic NDSRIs remain unstudied	[144]
Voltammetric/conductive filament optimization	PLA/graphite and PLA/carbon black filaments	LOD: 0.07 µM (uric acid), 0.11 µM (dopamine)	Printable composites function as transducers, but molecular selectivity requires added MIP or aptamer recognition layers	[143,145]
Voltammetric/sustainable conductive fillers	PLA/biochar conductive filament	LOD: 0.01 µM (carbendazim); Rct reduced from ~9.1 kΩ to ~1.0 kΩ	Green filaments improve sustainability and electrical performance, but pharmaceutical-matrix validation is still required	[148]
Voltammetric/MXene electron-transfer kinetics	Ti_3_C_2_T_x_ MXene nanoelectrodes	k^0^ ~ 0.03 cm s^−1^ (5× higher than V_2_CT_x_)	Provides mechanistic basis for fast signal transduction in ultra-trace sensing of tricyclic NDSRIs	[149]
Voltammetric/MXene aptasensor benchmark	Ti_3_C_2_T_x_-AuNP aptasensor (chloramphenicol)	LOD: 0.03 pM	MXene interfaces support ultra-sensitive aptamer readout, though chloramphenicol is less bulky than tricyclic NDSRIs	[150]
Voltammetric/MXene heterostructure sensing	Ti_3_C_2_T_x_ heterostructures (ciprofloxacin, etc.)	Sub-nanomolar to picomolar detection	Confirms high analytical sensitivity, but recognition selectivity must be engineered via MIP or aptamer layers	[150,151]
Capacitive/MXene restacking control	Crumpled MXene inks	Interlayer spacing: 1.35 → 1.62 nm; 8.2 F cm^−2^	Larger gallery spacing improves accessible surface area, preventing restacking bottlenecks for sterically constrained tricyclic analytes	[152,153]
Voltammetric/spacer-assisted MXene architectures	MXene composites with carbonaceous spacers	Salt removal: 25.82 mg g^−1^; 98.1% retention	Spacer insertion reduces pore-mouth blocking and improves interlayer accessibility for bulky NDSRIs	[151,154]
Voltammetric/porous MXene sensing architecture	Breath-figure porous MXene film	LOD: 0.17 µM (dopamine)	Porous MXene improves accessibility for organics, but sensitivity remains lower than optimized aptasensors	[155]
Conductometric/oxidation stabilization	Sodium ascorbate-capped MXene	92% conductivity retained after 80 days	Mitigates MXene oxidation for practical sensor storage and shelf-life deployment	[153]
Capacitive/oxidation-resistant 3D MXene/carbon	3D porous MXene/graphene architecture	94% capacitance retention after 20,000 cycles	Carbon-hybrid 3D architectures preserve electrochemical function, though selectivity depends on receptor engineering	[153,156]

**Table 2:** Small-molecule aptamer discovery constraints and computational rescue strategies for N-CZN-like targets.

Aptamer-Discovery Strategy	Reported Evidence/Performance Metric	N-CZN-Specific Relevance	Design Implication/Limitation	References
Capture-SELEX/small-molecule SELEX burden	8–20 rounds for DNA; 6–12 rounds for RNA; 2–3-month process time; Target-specificity achieved at cycle 8 with Kd ~200 nM; 60 nt randomized region.	Indicates slow and inefficient enrichment for tricyclic nitrosamines	Round burden alone makes purely empirical selection unattractive for a hard small-molecule target	[164,174]
Biased library strategy	Reported up to 99% probability of finding binders despite 10,000-fold reduction in diversity	Shows that rational library design can dramatically improve success probability	Strong argument for non-randomized N-CZN-focused library design	[173]
Nuclease-assisted SELEX	Nanomolar binders to AB-FUBINACA obtained in five rounds, with affinities 25–100-fold tighter than conventional selection after 11 rounds	Shows that optimized small-molecule selection can materially outperform classical SELEX	One of the strongest empirical arguments for using engineered SELEX formats in this review	[169]
CE-SELEX limitation	1–4 rounds yield Kd 17.41–29 nM for proteins, but negligible	Relevant because N-CZN–DNA complexes are also likely to show subtle hydrodynamic and charge changes	CE-SELEX is elegant but likely too weakly resolving for this target class without additional engineering	[170,171,172]
Nitrosamine stereochemical complexity	Z/E shifts reported around 10–60%; stabilization differences around 5 kcal/mol	Confirms that N-CZN should not be modeled as a single rigid ligand	Ignoring E/Z isomeric states would be a major methodological weakness in N-CZN aptamer design	[192,193]
Consensus docking improvement	Spearman correlations up to 0.89 with experimental trends in stereochemically sensitive ligand ranking	Supports weighted multi-isomer docking for N-CZN	Strong support for multi-engine, multi-state docking rather than single-score ranking	[199]
Aptamer binding-mode evidence	NOE-derived aptamer–ligand contacts in the AFB1 system clustered around approximately 2–5.5 Å	Supports a compact stacked aromatic-binding cavity model for N-CZN	Structural data strongly favor π-stacking-centered recognition rather than purely hydrogen-bond-driven models	[200]
ATP–aptamer energetic decomposition	Binding is dominated by dispersion/π-stacking (distances 2.0–5.5 Å) rather than by single hydrogen bonds.	Highly relevant to a tricyclic aromatic nitrosamine scaffold	Supports designing for aromatic surface complementarity first, with nitroso hydrogen bonding as a secondary specificity element	[202,203]
MD ensemble docking advantage	Using >1 conformer (multiple states) improved pocket identification compared with a single lowest-energy structure.	Important because ssDNA aptamers are conformationally heterogeneous	Makes ensemble-based docking essential rather than optional for N-CZN aptamer screening	[181,183]
Computational ranking/maturation	AFB1 case: best variant identified by MM-PBSA; ΔΔG trends mirrored experimental ranking binding energies ranged from −30.27 to −32.25 kcal/mol	Shows that in silico maturation can rank useful candidates before wet-lab screening	Strong support for pre-SELEX or post-SELEX computational triage	[184]
Truncation protocol utility	APTAMD-supported truncation of 20–35% without loss of affinity ML-derived truncations 70% shorter with Kd of 1.5 nM	Suggests candidate N-CZN aptamers can later be shortened for better surface performance	Especially relevant for electrochemical sensors, where shorter probes may reduce steric crowding and improve electron-transfer efficiency	[179]
Deep learning aptamer mining	DeepAptamer recovered high-affinity sequences from weakly enriched early rounds ML predicted high-affinity DNA aptamers at a rate 11-fold higher than random perturbation	Useful for rescuing signal from low-efficiency small-molecule selections	Major advantage for difficult targets where classical enrichment is poor	[208]
AI classification performance	AUROC near 0.998 and classification accuracy near 0.995 reported in aptamer-related models	Suggests sequence ranking and non-binder filtering can be strongly automated	These tools can support prioritization, but target-specific experimental confirmation remains essential	[189,190,191]

**Table 3:** Signal-off aptasensing as a transduction strategy for bulky tricyclic NDSRIs: performance metrics and translational relevance.

Signal-Off Architecture	Model Target/Matrix	Reported LOD	NDSRI-Specific Relevance
MXene-AuNP electrochemical aptasensor	Chloramphenicol/Honey	0.03 pM	Steric + electrostatic blocking of small organic analyte [120]
MXene paper-based label-free aptasensor	Aflatoxin B1/Corn	0.04 ng/mL	Bulky aromatic target; impedimetric signal-off via reduced electron transfer [119]
Ratiometric PEC-EC aptasensor	Streptomycin/Buffer	10 nM [215]	Signal restored when steric hindrance removed; direct proof crowding governs response [215]
MXene-OMC aptasensor	Aminoglycosides/Milk	3.51 nM	Significant DPV signal decline from aptamer–analyte biochemical reactions [217]
Folding-based SWV aptasensor	SARS-CoV-2 Spike Protein	100 fM	Steric hindrance works with tethered redox labels; relevant for N-CZN platform strategies [145]
V2CTx MXene SWV aptasensor	HER-2 Biomarker	0.36 ng/mL	MXene surface-to-volume ratio enables efficient aptamer immobilization and signal enhancement [218]
Carbon nanodots impedimetric aptasensor	17β-Estradiol/River water	0.5 pM	Folded aptamer–target complex alters electrostatic repulsion and redox species access [219]
Ferrocene-labeled EIS/SWV aptasensor	Soluble KIT/Human serum	1.15 pg/mL	Conformation-changing aptamer; demonstrates signal-off viability in complex biological matrices [220]
OMC-Au nano steric hindrance aptasensor	VEGF	1 ng/L	Binding forms steric hindrance increasing charge-transfer resistance at the interface [221]
Dual-aptasensor (AFP channel)	Alpha-fetoprotein/Serum	1.77 pg/mL	Aptamer–protein complex induces steric hindrance, suppressing redox signals [222]

**Table 4:** Experimental evidence defining the multi-NDSRI “mixture dilemma” in clozapine electrochemical QC.

System/Analytical Issue	Key Finding	Quantitative Result	Analytical Implication	Reference
Clozapine NDSRIs (N-CZN + N-CZNC)	Simultaneous LC-MS/MS quantification validated in API and formulations	R^2^ > 0.999; N-CZN LOD/LOQ 0.058/0.177 µg mL^−1^; N-CZNC 0.014/0.044 µg mL^−1^; recoveries 88–102%	Confirms real co-occurrence and need for dual monitoring	[223]
Ciprofloxacin multi-NDSRI method	Three NDSRIs plus other nitroso impurities monitored together	Trace-level simultaneous LC-MS/MS method reported	Multi-NDSRI control is already required in other APIs	[228]
Moxifloxacin nitrosamines	Simultaneous N-MOX and DNPP quantification	Eco-friendly LC-TQ-MS/MS reported for tablets and eye drops	Supports co-monitoring framework for related APIs	[229]
Clonidine nitrosamines	Multiple nitrosamine impurities quantified together	N-nitroso and N-dinitroso clonidine measured in drug substance/tablets	Reinforces regulatory need for multi-impurity control	[230]
Peak overlap theory	Overlapped voltammetric peaks can be resolved only computationally	Resolution possible down to ~2.5 mV separation with Kalman filtering	Raw voltammograms of similar analytes are insufficient	[232]
Acid mixture SWV	One peak decomposed into overlapping species	Gaussian deconvolution enabled simultaneous quantification	Direct precedent for deconvolving merged electrochemical peaks	[233]
Tocopherol isomers	Similar analytes remained poorly separated electrochemically	LOD 0.89–3.72 × 10^−6^ mol L^−1^; recoveries 72–128.5% after deconvolution	Similar analogs require mathematical separation	[234]
Levamisole in cocaine	Hidden overlapping peak recovered by deconvolution	Oxidation peaks at +0.85 V and +0.96 V; electrochemical/reference agreement r = 0.995	Strong analog for closely overlapping NDSRIs	[235,236]
Dihydroxybenzene isomers	FFT-SWV + chemometrics resolved ternary overlap	RMSEV 0.042–0.066 µM; LOD ~0.045–0.046 µM	Chemometrics can recover multicomponent information from composite peaks	[237]
Nitroaniline isomers	Wavelet/PLS-based chemometrics improved prediction	Best RSEP among tested models	Advanced preprocessing improves separation of severe overlaps	[238]
SWV interference control	Sampling-window optimization can separate kinetic contributions	Selective enhancement/suppression of analyte vs interferent signals	Useful for partially resolving similar redox responses	[240]
Advanced SWV-derived methods	New protocols provide richer structured data	Potential-corrected, multi-sampled, cumulative SWV and Faradaic spectroscopy reviewed	Better input for post hoc deconvolution of NDSRI mixtures	[239]

**Table 5:** Benchmark analytes mapped to projected N-CZN risk.

Benchmark Analyte	Platform/Recognition	Matrix	Reported LOD	Structural Read-Across to N-CZN	Expected N-CZN Difference	Direction of Risk
Chloramphenicol (CAP); smaller antibiotic, aptamer-compatible	Ti_3_C_2_T_x_-AuNP aptasensor [120]	Honey	0.03 pM	Useful for MXene signal gain, but CAP is explicitly smaller and less bulky than tricyclic NDSRIs	N-CZN should bind in a more sterically hindered, slower-transfer regime	Underestimation of difficulty
Chloramphenicol (CAP); dual-recognition benchmark	MIP–aptamer electrochemical sensor [12]	Not specified in abstract	3.3 × 10^−9^ g/L	Supports hybrid-recognition benefit and signal-off blocking	N-CZN should be harder because analyte bulk and pharmaceutical background are greater	Underestimation
Chloramphenicol (CAP); embedded aptamer/MIP	Aptamer–MIP impedimetric sensor [277]	Milk	0.3 pM	Strong benchmark for dual-recognition cavity-plus-aptamer design	N-CZN likely shows more nonideal cavity access and stronger matrix masking	Underestimation
AFB1; rigid aromatic toxin	Hybrid aptamer–MIP electrochemical sensor [287]	Milk	12.0 pg/L	Better rigidity match than CAP because AFB1 is also a rigid aromatic small molecule	N-CZN still faces added pharmaceutical excess-ratio interference from clozapine/N-CZNC	Moderate underestimation
AFB1; anti-interference benchmark	Aptamer–MIP with COF/Au-Pt interface [282]	Complex food matrices	2.31 × 10^−4^ ng/mL	Strong evidence that dual recognition helps when single-mode sensors give false positives	N-CZN should benefit similarly from pre-enrichment plus precise recognition	Mechanistic support, moderate risk
AFB1; MXene aptasensor	Ti_3_C_2_T_x_/cDNA-PAA aptasensor [288]	Peanuts	1.0 × 10^−3^ ng/L	Supports MXene-enabled sensitivity and stability	N-CZN may show lower net gain if oxidative drift and pore blocking offset signal amplification	Possible overprojection of sensitivity
NDMA; small flexible nitrosamine	MIP electrochemical nanosensor [24]	Serum and tap water	3.63 fM	Nitrosamine chemistry is directly relevant	N-CZN is much more rigid, bulky, and matrix-suppressed than NDMA	Strong underestimation
NDMA; reversible MIP benchmark	Dual-monomer MIP on electroactive layer [23]	Aqueous environments/water samples	1.16 ppb	Useful for reversibility and indirect blocking readout	N-CZN regeneration could be worse because bulkier interfacial binding can promote irreversible fouling	Underestimation of regeneration risk
NDEA; small flexible nitrosamine	Electropolymerized MIP electrochemical sensor [158]	Commercial serum	5.92 fM standard, 12.60 fM serum	Direct nitrosamine comparator with biological interferents [158]	Easier than N-CZN because NDEA lacks fused-ring rigidity and parent-API scaffold overlap	Strong underestimation
NDPhA/NDEA; aromatic vs aliphatic nitrosamines	PtNPs/MWCNTs/MOF electrochemical sensor [160]	Tap water and tobacco	6.534 × 10^−3^ μg/mL for NDPhA; 0.173 μg/mL for NDEA	Shows analyte-dependent detectability even within nitrosamines	N-CZN may behave closer to the more hydrophobic aromatic nitrosamine than to small dialkylnitrosamines	Mixed, but still harder overall
Eight standard nitrosamines	Validated LC-MS/MS reference method [289]	API and capsule matrices	0.154–0.560 ng/mL DL	Provides pharmaceutical-matrix trace benchmark	Attached review notes tricyclic NDSRIs create a larger performance gap than standard nitrosamines	Underestimation

## Data Availability

No new data were created or analyzed in this study. Data sharing is not applicable to this article.
